# Printable Aligned Single-Walled Carbon Nanotube Film with Outstanding Thermal Conductivity and Electromagnetic Interference Shielding Performance

**DOI:** 10.1007/s40820-022-00883-9

**Published:** 2022-09-01

**Authors:** Zhihui Zeng, Gang Wang, Brendan F. Wolan, Na Wu, Changxian Wang, Shanyu Zhao, Shengying Yue, Bin Li, Weidong He, Jiurong Liu, Joseph W. Lyding

**Affiliations:** 1 Key Laboratory for Liquid-Solid Structural Evolution and Processing of Materials, Ministry of Education, School of Materials Science and Engineering, Shandong University, Shandong, Jinan, 250061 People’s Republic of China; 2 Department of Electrical and Computer Engineering, Beckman Institute for Advanced Science and Technology, University of Illinois at Urbana-Champaign, Urbana, IL 61801 USA; 3 Department of Chemistry, Swiss Federal Institute of Technology in Zurich (ETH Zürich), 8092 Zurich, Switzerland; 4 School of Materials Science and Engineering, Nanyang Technological University, 50 Nanyang Avenue, Singapore, 639798 Singapore; 5 Empa, Swiss Federal Laboratories for Materials Science and Technology, Überland Strasse 129, 8600 Dübendorf, Switzerland; 6 Institute for Advanced Technology, Shandong University, Jinan, 250061 People’s Republic of China

**Keywords:** Aligned film, Single-walled carbon nanotube, Lightweight, Flexible, Thermal conductivity, Electromagnetic interference shielding

## Abstract

Ultrathin, lightweight, and flexible aligned single-walled carbon nanotube (SWCNT) films are fabricated by a facile, environmentally friendly, and scalable printing methodology. The aligned pattern and outstanding intrinsic properties render “metal-like” thermal conductivity of the SWCNT films, as well as excellent mechanical strength, flexibility, and hydrophobicity. Further, the aligned cellular microstructure promotes the electromagnetic interference (EMI) shielding ability of the SWCNTs, leading to excellent shielding effectiveness (SE) of ~ 39 to 90 dB despite a density of only ~ 0.6 g cm^−3^ at thicknesses of merely 1.5–24 µm, respectively. An ultrahigh thickness-specific SE of 25 693 dB mm^−1^ and an unprecedented normalized specific SE of 428 222 dB cm^2^ g^−1^ are accomplished by the freestanding SWCNT films, significantly surpassing previously reported shielding materials. In addition to an EMI SE greater than 54 dB in an ultra-broadband frequency range of around 400 GHz, the films demonstrate excellent EMI shielding stability and reliability when subjected to mechanical deformation, chemical (acid/alkali/organic solvent) corrosion, and high-/low-temperature environments. The novel printed SWCNT films offer significant potential for practical applications in the aerospace, defense, precision components, and smart wearable electronics industries.

**Supplementary Information:**

The online version contains supplementary material available at 10.1007/s40820-022-00883-9.

## Highlights

- Ultrathin, lightweight, and ultraflexible aligned single-walled carbon nanotube (SWCNT) films were fabricated via a facile, environmentally friendly, and scalable printing methodology.
- The aligned pattern and outstanding intrinsic properties of SWCNTs rendered “metal-like” thermal conductivity, excellent mechanical strength, hydrophobicity, and remarkable electromagnetic interference (EMI) shielding performance of the films.
- The excellent EMI shielding stability and reliability when subjected to mechanical deformation, chemical corrosion, and extreme environments demonstrated the significant potential of the films in aerospace, defense, and smart wearable electronics.

**Supplementary Information:**

The online version contains supplementary material available at 10.1007/s40820-022-00883-9.

## Graphical abstract

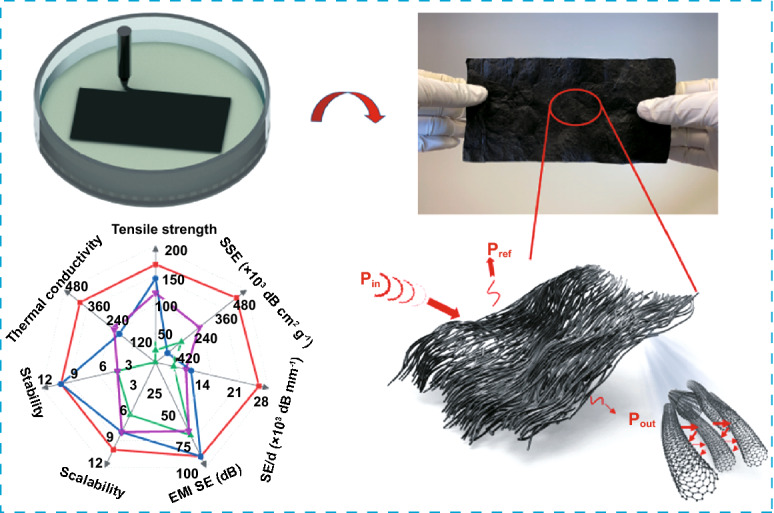

## Introduction

With the continuing reliance and proliferation of electronics, increased failure of electronic equipment and potential health impacts due to greater electromagnetic interference (EMI) and pollution is inevitable [1–5]. The urgent need for a solution in the form of high-performance EMI shielding materials is made particularly clear by the development of high-frequency, high-speed, fifth generation (5G) and inevitable sixth-generation (6G) technologies. An ideal shielding material would integrate superb shielding properties with high thermal conductivity, mechanical strength, flexibility, reliability, and durability even in extreme environments achieved while maintaining thin layers and a low density [6–13]. Nanomaterials, such as metal nanowires or nanofibers [3, 14], transition‐metal carbides (MXene) layers [1, 15, 16], graphene sheets [17, 18], or carbon nanotubes (CNTs) [10, 19], all demonstrate the large specific surface areas and outstanding electrical, mechanical, and thermal properties that suggest the potential for being able to satisfy the growing need for high-performance shielding macrostructures. Recently, the most impressive shields involved the vacuum-filtrated or blade-coated freestanding MXene films [6, 20], metal nanofibers-wrapped polymer nanofibers mats, or graphene films [14, 17, 21]. These materials exhibited high EMI shielding effectiveness (SE) even at ultralow thicknesses in the range of a few to tens of micrometers. Their normalized specific SE (SSE) [6, 22], defined as the SE divided by the thickness and density of materials to describe the lightweight high-performance EMI shields, can reach breakthrough values between 37 332 and 232 860 dB·cm^2^ g^−1^ significantly surpassing that of other materials [6, 17, 20, 21, 23]. Nevertheless, either reduced reliability in harsh environments, such as in the presence of acids, or at low/high temperatures or requirements for complex and environmentally hazardous chemical treatments limit the scalability and performance of these novel shielding macrostructures. More efficient, convenient, green, and scalable preparation techniques to facilitate research into more reliable and high-performance EMI shielding materials are highly desired.

Compared with conventional metal shields or most metalliferous nanomaterials, carbon nanomaterials exhibit tremendous advantages as emerging EMI shielding materials thanks to low-cost mass production, low density, excellent mechanical flexibility, chemical stability, and good reliability in varied circumstances [7, 10, 17–19, 23–26]. However, the difficulty in processing or dispersing these carbon nanomaterials due to their chemically inert surfaces makes the construction of nanocarbon-based macrostructures challenging [2, 18, 22, 27]. Furthermore, the electrical conductivity, a primary parameter that determines the EMI shielding performance [2, 6, 28], of these architectures is still too low to achieve the high EMI shielding performance promised by the intrinsic electrical conductivity of nanocarbon. This discrepancy arises from the relatively low derived conductivity of the employed macrostructures caused by disorder and agglomeration of the nanoparticles that occur during the assembly process [14, 22, 27]. For instance, the most commonly used multi-walled CNT (MWCNT)-based shields usually involve difficult dispersion processes that yield limited conductivity and a large contact resistance due to the disordered winding or aggregation of the one-dimensional (1D) tubes [29, 30]. Similarly, graphene oxide (GO), a derivative of graphene, has been widely demonstrated as a precursor for graphene-based shields. However, the electrically insulating GO has to be reduced to a conductive architecture and the electrical conductivity of reduced GO is still limited [23, 31]. Due to these limitations, the EMI shielding performance of the carbon-based shields compares poorly in comparison to that of metal nanomaterials or recently developed MXene competitors [6, 8, 14, 16, 32]. In contrast, single-walled CNTs (SWCNTs) have been widely studied and exhibit peak electrical conductivity exceeding other carbon nanomaterials due to their electronic structure derived from the carbon *sp*^2^ lattice hybridization [19, 26, 33]. Despite the increased difficulty in dispersing SWCNTs due to their ultrathin nature as compared to MWCNTs, a superior EMI shielding performance is predicted for SWCNTs [34–38]. Presently, either suspension-based deposition such as vacuum filtration or chemical vapor deposition (CVD) methods are employed to prepare freestanding SWCNT films [39, 40]. The former usually requires expensive supporting substrates and energy-intensive low vacuum conditions. Moreover, freestanding films are difficult to separate from the preparation substrates and thus suffer severe limitations in terms of production efficiency and product quality [41, 42]. Similarly, CVD is an energy- and time-consuming process with poor control of the resulting film’s size (thickness and width) [2, 39]. Efficient and scalable preparation of SWCNT films for high-performance EMI shielding macrostructures would be highly useful but remains out of reach for widespread adoption in the booming electronic field [26, 43]. The growth of industries such as the internet of things creates significant demand for a material that, in addition to EMI shielding performance, also demonstrates impressive properties such as miniaturization and integration potential, excellent electrical and thermal conductivities, good hydrophobicity, high resistance to mechanical deformation, and reliability in extreme environments all while remaining ultrathin and lightweight. SWCNTs have the great potential to satisfy all of these requirements regardless of the challenges in preparation of macrostructures with a rational design of microstructure.

Here, we report a simple, facile, environmentally friendly, and scalable wet-spinning-based printing method to construct lightweight, ultrathin, and flexible freestanding aligned SWCNT films. The intrinsically excellent properties of SWCNTs and the aligned pattern in the films endow the low-density SWCNT films with excellent electrical conductivity and thermal conductivity comparable to metals [33, 44–46]. In addition, these films demonstrate excellent mechanical strength, flexibility (bendability, rollability, and foldability), and good hydrophobicity including waterproof behavior. Their aligned cellular microstructure of the film enables the macroscopic expression of the intrinsic SWCNT shielding ability. Our aligned SWCNT films reach EMI SE values of ~ 39–90 dB with thicknesses of merely 1.5–24 µm, respectively, at a density of ~ 0.6 g cm^−3^. The lightweight SWCNT films also demonstrate high EMI SE of more than 54 dB even in an ultra-broadband frequency range of around 400 GHz at a thickness of 8.0 µm. The ultrathin and lightweight nature of these films results in thickness-specific SE (SE/*d*, namely SE divided by thickness) of up to 25,693 Db mm^−1^ and an unprecedented SSE of 428 222 dB·cm^2^ g^−1^, significantly surpassing the abilities of other shielding materials. Moreover, these films exhibit excellent EMI shielding stability and reliability even when subjected to mechanical deformation, chemical (acid/alkali/organic solvent) corrosion, and high-/low-temperature treatments. These results demonstrate the immense potential of our ultrathin, flexible, and lightweight SWCNT films, along with customized fabrication processes, for practical EMI shielding applications in aerospace, defense, portable electronics, and smart wearables industries.

## Experimental

### Materials and Methods

#### Printing Fabrication of Freestanding SWCNT Film

Raw SWCNT (diameters of ~ 1.6 nm and length of ~ 5 µm) powders are commercially purchased from *OCSiAl*. To remove the impurities in the raw materials, SWCNT powders were purified before use. During the purification process, raw SWCNTs were mixed with hydrochloric acid and H_2_O_2_ and heated to 60 °C for 4 h. SWCNTs were then collected over a filter paper after washing with DI water and dried in air. To disperse purified SWCNTs in water, 50 mg SWCNTs and 450 mg sodium dodecyl sulfate (SDS) were mixed in 25 mL deionized (DI) water through 5 min ultrasonic bath followed by 30 min tip-sonication treatment (Sonifier S250). The obtained SWCNT dispersion was then loaded into a syringe pump system mounted onto a 3D printer frame, enabling precisely controllable syringe (with the nozzle of flat-tip needle, gauge size: 19) movement during printing. SWCNT dispersion was then continuously extruded onto the silicone rubber substrate in isopropyl alcohol (IPA) bath with directionally moving of syringe. The moving path of the syringe was controlled by the programmed 3D printer through pre-loaded codes (Video S1). Once the printing process was completed, IPA liquid was drained away with a pipette and the printed SWCNT film was left on the rubber substrate for drying. After peeling off from the substrate, the freestanding SWCNT film was obtained. As-prepared SWCNT films usually contained some residues of surfactant. To remove the surfactant residues, the as-prepared films were immersed in DI water and washed several times. Moreover, we demonstrate the capability of integrating magnetic iron oxide nanomaterials into the SWCNT film. Herein, iron nitrate crystals were dissolved into IPA to make coagulation solution (the concentration of iron nitrate was 0.01 mol L^−1^). Then, the obtained SWCNT dispersion was continuously extruded into the coagulation bath through the same printing fabrication as the raw SWCNT film. The metal nitrate which was previously dissolved in coagulation solution would infiltrate with fast exchanging process and uniformly coated onto well-dispersed carbon material. Finally, the obtained SWCNT/iron nitrate composite was transferred to a tube furnace and annealed in argon atmosphere at 250 °C for 3 h followed by the annealing at 300 °C with hydrogen flow for 3 h. Because of the thermal decomposition of metal nitrates under annealing, iron nitrate would be converted to iron oxide and SWCNT/magnetic iron oxide hybrid was prepared.

### Characterization

Microstructures of as-prepared freestanding SWCNT films were characterized by Field-Emission SEM (FEI Quanta FEG 450 and Hitachi S4800). Raman spectra were acquired by using Horiba Raman Confocal Imaging Microscope (LabRAM HR 3D) with a 532 nm laser. 4-terminal measurements were used to test as-prepared SWCNT film’s electrical conductivity through Keithley 4200 semiconductor characterization system. The pressure drop between top and bottom surfaces of aligned SWCNT film was measured by a pressure transducer (PC409, Omega, Inc.). SAXS and WAXS measurements were conducted on a Xeuss 3.0 system from Xenocs. Cu target of 30 W optical tube power and 30 µm focal spot diameter were used. These measurements used an Eiger2R 1 M detector. Measurements were done in transmission mode using a sample to detector distance of 60 mm and an exposure time of 600 s in the *q*-range: 2θ_min_ ≤ 0.013º, *q*_min_ ≤ 0.012 nm^−1^, 2θ_max_ ≥ 75º, *q*_max_ ≥ 49 nm^−1^. Mechanical properties of as-prepared freestanding SWCNT film were characterized by T.A. Systems Q800 Dynamic Mechanical Analyzer. For the tensile stress measurements, SWCNT film was fixed onto a hollow paper frame with gauge length of 10 mm and width of 4 mm and then placed into the test apparatus with clamps. The paper frame was cut before the dynamic mechanical measurement. Stress–strain curves were obtained under a controlled force model with ramp force rate of 0.2500 N/min. All measurements were performed at room temperature. The Young’s modulus was determined from the slope of the linear region of the stress–strain curves. Theoretical simulation and experimental measurement of SWCNT’s thermal conductivity, and the EMI shielding performance test and calculation are included in the supporting information.

## Results and Discussion

### Preparation, Structure, and Properties of SWCNT Film

**Fig. 1:**
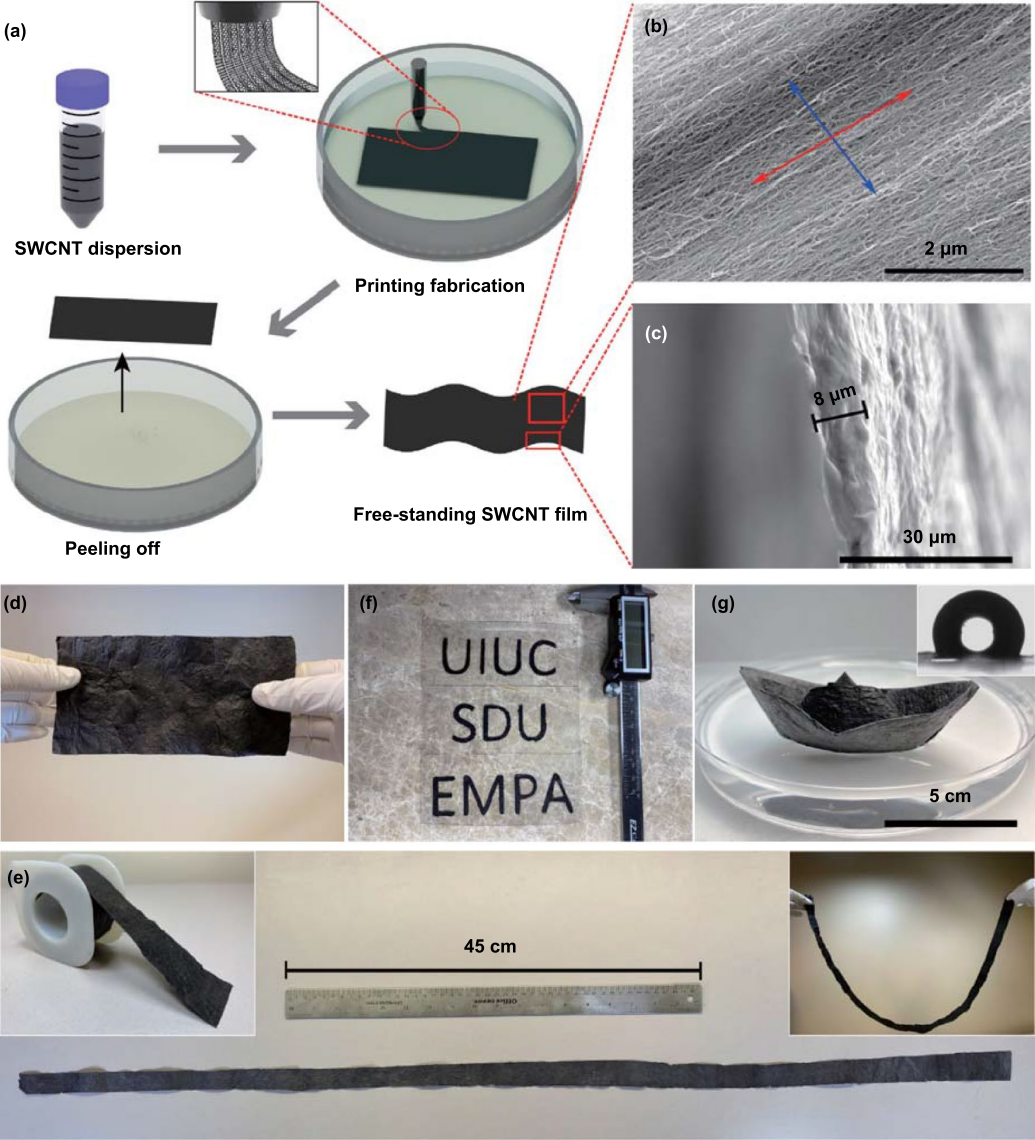
**a** Schematic for printing fabrication process of freestanding SWCNT film. **b** In-plane SEM image of the SWCNTs, showing the aligned SWCNTs in print (red arrow) direction. **c** Side-view SEM image of the SWCNT film, showing a typical thickness of 8 µm. **d** Photograph of a large-area (10 × 20 cm^2^) freestanding SWCNT film held by hands. **e** Demonstration of a meter-long freestanding SWCNT film. **f** Printed letters of SWCNTs on PET. **g** Origami boat made from SWCNT film floating on water (inset shows the water contact angle of 125° for the film)

The printing fabrication process of the freestanding SWCNT films is schematically illustrated (Fig. 1a, Video S1). The purified SWCNT powders were dispersed in deionized water through an ultrasonication process [42, 47]. Afterward, the well-dispersed SWCNT dispersion was loaded into a syringe pump mounted onto a home-made 3D printer system. A 3D printer system and software enabled precisely controlled movements and path planning of the syringe during printing. During printing, the SWCNT dispersion was continuously extruded onto a silicone rubber substrate in an isopropyl alcohol (IPA) bath. The dragging force caused by the movement of the syringe through the IPA resulted in the formation of aligned SWCNT bundles in the coagulation bath. The 3D printer system allowed for rapid and facile adjustments to the size and configuration of the resultant films. The weak interaction between SWCNT and rubber made it feasible to separate the SWCNT film from the substrate after drying. The aligned microstructure of the SWCNT films is observed along the motion direction of the nozzle (red arrow) during printing (Fig. 1b). Moreover, by adjusting the number of printings passes the film width (blue arrow dimension), and thicknesses of the SWCNT film are easily controlled. A typical, large-area (10 × 20 cm^2^) SWCNT film with a thickness of 8 µm is exhibited (Fig. 1c–d), and the thickness of mechanically robust freestanding films can reach down to 1.5 µm (Fig. S1a). A meter-long freestanding and flexible SWCNT film demonstrates the potential for scalable production (Fig. 1e). In addition to printing aligned SWCNT films by simple movements of the nozzle, this programmable printing process has the capability of constructing complex patterns and advanced architectures. In order to demonstrate the tremendous potential of the printed SWCNT films in practical applications requiring custom-designed shapes and sizes, the affiliated universities are represented by their respective acronyms printed on transparent polyethylene terephthalate (PET): UIUC (University of Illinois at Urbana-Champaign), SDU (Shandong University) and EMPA (Swiss Federal Laboratories for Materials Science and Technology) (Fig. 1f).

The outstanding intrinsic properties of the SWCNTs combined with the neoteric fabrication process lead to interesting emergent properties in the resulting films. Due to their ordered microstructure, the freestanding SWCNT films are incredibly robust when bent, rolled, and folded (Fig. 1e). An origami boat made from the film floating on water is shown in Fig. 1g, suggesting as-prepared SWCNT film’s lightweight and hydrophobic nature (Fig. S2a). As moisture is an important parameter affecting the stability, reliability, and durability of electronics, the demonstrated hydrophobicity increases the film’s auspiciousness [8, 14]. A water contact angle as high as 125° has been observed for the SWCNT film, which lends excellent waterproof capability. The SWCNT film can maintain its original freestanding shape and structural integrity even after immersion in water for fifteen days followed by an additional ten-minute sonication treatment **(**Fig. S2b–c, Video S2). Despite this hydrophobicity, a porosity of ~ 60% (Note S1 of Supporting Information) for the low-density SWCNT films (~ 0.6 g cm^−3^) allows for high air-permeability such that the pressure drop across the cellular SWCNT macrostructures is lower than that of commercial N95 masks (Fig. S3). These properties, combined with the facile and scalable printing methodology (which is capable of integrating additional functional additives such as magnetic materials (Fig. S4)), make the SWCNT films a promising prospect for the design and production of multifunctional smart electronics or textiles.

The synchrotron X-Ray measurements were used to quantitatively verify the orientation structure of SWCNT films. As shown in Fig. S5a–b, the small- and wide-angle X-Ray scattering (SAXS/WAXS) patterns of the printed aligned SWCNT films showed the presence of a broad elliptical halo. In general, elongated broad elliptical halos were caused by the improved oriented structures of the films. In contrast, random SWCNT buckypaper prepared from vacuum filtration of the same raw material was also characterized. For the random films, both SAXS and WAXS patterns show circular and uniform annular halo (Fig. S5c–d), indicating that the internal structure of random SWCNT film was isotropic. Moreover, the improved orientation could also be observed from the variation trend of azimuthal-integrated intensity distribution curves from SAXS and WAXS patterns (Fig. S5e–f). The constant intensity values at different azimuthal angles of the random SWCNT films show that there was no obvious structural ordering. In addition, the degree of orientation (*f*_c_) of the printed SWCNT film calculated from azimuthal-integrated intensity distribution curves of SAXS and WAXS was 0.54 and 0.67, respectively. The above measure results show that the printing method rendered a considerable orientation of the SWCNT films.

**Fig. 2:**
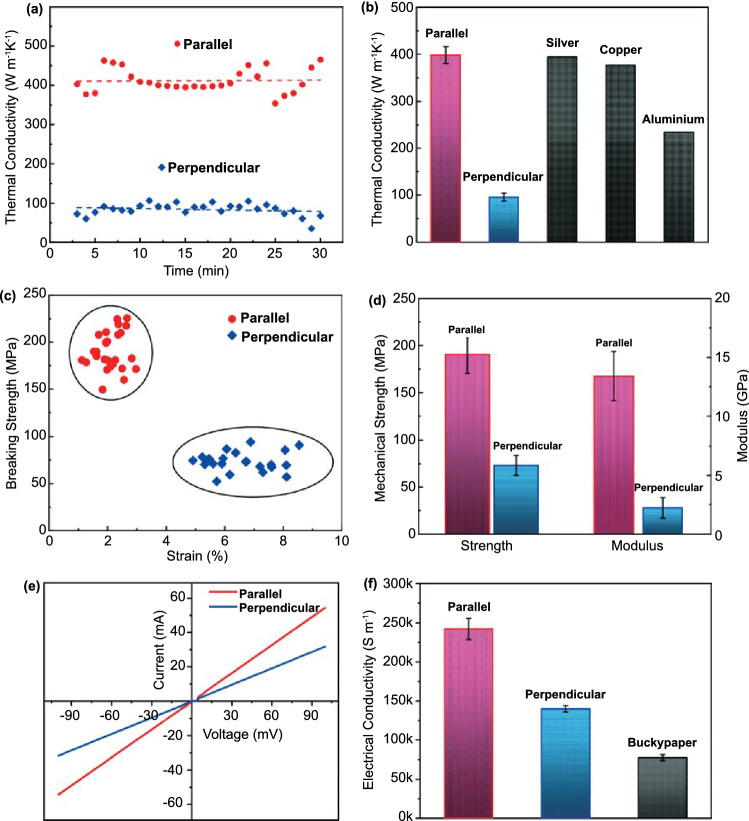
Properties of as-prepared freestanding SWCNT film in parallel and perpendicular orientations. **a** Typical measured thermal conductivity and **b** average thermal conductivity of SWCNT film in parallel and perpendicular directions, as well as some tested pure metal plates (silver, copper, and aluminum). **c** Scatter plots of as-prepared films’ tensile mechanical property measured and **d** average tensile strength and Young’s modulus of SWCNT films in parallel and perpendicular directions. **e** Typical *I–V* curves of SWCNT film and **f** average electrical conductivity of aligned SWCNT film in parallel and perpendicular directions as comparison to the random network SWCNT buckypaper

High thermal conductivity is important for the continued progress of electronics due to the demands of rapidly developing high-speed communication and signal processing technologies [17, 31]. To indicate the thermal conductivity theoretically, the lattice thermal conductivities of armchair SWCNTs (8, 8) and (12, 12) were investigated by solving the phonon Boltzmann transport equation (BTE) with the first-principles simulations (details included in the supplemental materials). The simulation results exhibited that individual SWCNT had a lattice thermal conductivity of more than 3000 W m^−1^ K^−1^ along the axial direction at 300 K (Fig. S6), which indicated its great potential in heat dissipation. The thermal conductivity of the as-prepared ultrathin SWCNT film was experimentally measured using a home-made apparatus (Fig. S7a–b); the films exhibit anisotropic thermal conductivities in parallel and perpendicular orientations with respect to the film’s alignment (print) direction (Fig. 2a). The thermal conductivity in the parallel direction is around 398 W m^−1^ K^−1^, which exceeds that of aluminum, copper, and even silver (Figs. 2b and S7c–d) and is about 140 and 2 times higher than that of MXene (2.84 W m^−1^ K^−1^) and graphene films (190 W m^−1^ K^−1^) [17, 48], respectively. The thermal conductivity in the perpendicular direction is ~ 96 W m^−1^ K^−1^. This yields an anisotropic thermal conductivity ratio greater than four, demonstrating the film’s potential as a material for thermal management devices [49–51]. Similarly, the tensile properties of the freestanding SWCNT films were investigated (Figs. 2c–d and S8) and found to be anisotropic. The parallel direction tends to have higher strength and lower breaking strain in comparison to the perpendicular direction, a further derivative of the aligned structure in the films. In the parallel direction, the SWCNT films have an average mechanical strength as high as 190.7 MPa and a Young’s modulus of 13.4 GPa; the perpendicular direction measures 73.3 MPa and 2.2 GPa, respectively. A comparison of the film’s thermal conductivity and mechanical strength against some contemporary materials (Table S1) demonstrates the potential of our aligned SWCNT film as an advanced macrostructure.

The remarkable intrinsic properties of SWCNTs combine with aligned microstructure in the as-prepared SWCNT films to generate remarkable electrical properties. Typical *I–V* curves for the SWCNT films were measured in both orientations using a standard four-probe technique (Fig. 2e). The average electrical conductivity in the parallel direction is 242,988 S m^−1^ compared with 140,102 S m^−1^ in the perpendicular direction. For further comparison, random network SWCNT buckypaper was prepared from a vacuum filtration of the same raw material and contrasted against the aligned SWCNT film (Figs. 2f and S9). Despite the high energy cost of the vacuum filtration process, the SWCNT buckypaper shows an inferior electrical conductivity of 77,323 S m^−1^, which is attributed to the disordered network of SWCNT bundles. It’s worth noting that the electrical conductivity of SWCNT buckypaper is even lower than that of the aligned film in the perpendicular direction, which can be attributed to the low density (~ 0.3 g cm^−3^) of the buckypaper as a result of filtration approach. Further, the organized pattern of the aligned SWCNT bundles in the printed films leads to a lower contact resistance across the entire plane of the macrostructure. Also, the stability and reliability of the electrical conductivity upon mechanical deformation (including bending, twisting, and kneading) are demonstrated (Fig. S10), proving the capability of our SWCNT films for ultraflexible electronics.

### EMI Shielding Performance of SWCNT Film

**Fig. 3:**
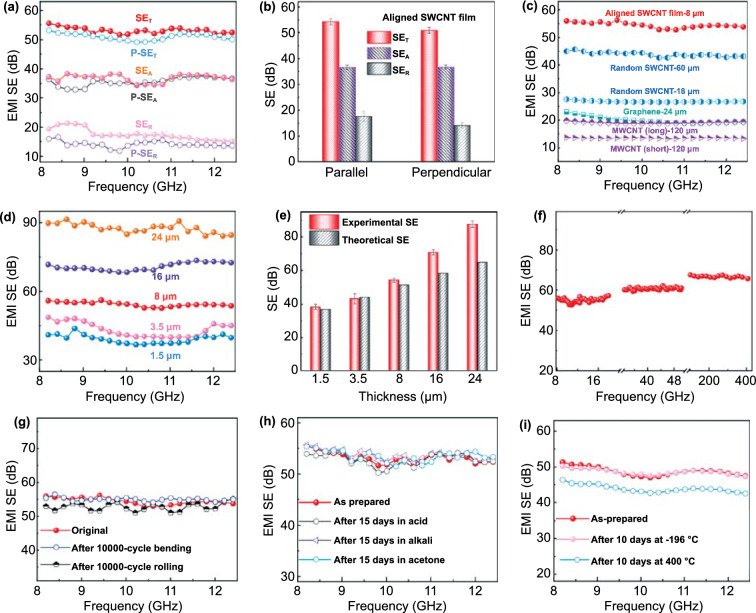
EMI shielding performance of the aligned SWCNT films. EMI Shielding performance **a** in the X-band and **b** at 10 GHz of the 8 µm thick aligned SWCNT films in different directions with respect to the electric field direction of incident EM waves. **c** EMI SE of aligned SWCNT films, and vacuum-filtrated random network SWCNT buckypaper, rGO film, and different aspect ratios of MWCNT buckypapers. **d** EMI SE in the X-band at various thicknesses for the SWCNT films. **e** Experimental EMI SE at 10 GHz of the aligned SWCNT films with various thicknesses, as comparison to the theoretical EMI SE of a homogeneous shield. **f** The experimental measured EMI SE values of the 8 µm thick aligned SWCNT films in ultra-broadband frequency range. **g** EMI SE of the films after 10,000 cycle bending and rolling in the whole X-band frequency range. EMI SE of the as-prepared SWCNT films **h** before and after strong acid, alkali, or acetone immersion treatments and **i** before and after low-/high-temperature treatments. The EMI SE is nearly unchanged after long-term storage in harsh environments, revealing a high chemical and structural stability of SWCNT film

The ultrathin, flexible, and lightweight SWCNT film provides outstanding EMI shielding performance due to the combination of the film’s excellent electrical conductivity, cellular structure, and aligned pattern. EMI shielding is a confluence of mechanisms including reflection (*SE*_R_), absorption (*SE*_A_), and multi-reflections, which result from mobile charge carriers, electric (or magnetic) dipoles, and multiple reflections (multi-reflections) of interior surfaces, respectively [2, 22, 27, 28, 52]. The primary parameter affecting the EMI SE is electrical conductivity. The inherent conductivity of SWCNTs and their aligned pattern in the film synergistically give rise to the excellent conductivity of the aligned SWCNT film, up to 242,988 S m^−1^, leading to an excellent X-band EMI *SE* of ~ 54 dB at a thickness of only 8 µm (Fig. 3a). This SE value corresponds to more than 99.999% attenuation of the incident EM waves, which is far beyond the industry-accepted *SE* value of 20 dB [2, 27]. The *SE*_A_ is higher than *SE*_R_ for the SWCNT film, which is consistent with the reported behavior for other carbon-based shielding architectures [2, 10, 14, 17, 53]. The EMI shielding performance (P-*SE*_T_, P-*SE*_R_, P-*SE*_A_) can also be tested in the case when the electric field direction of incident EM waves is perpendicular to the aligned SWCNTs. Compared with the shielding performance (*SE*_T_, *SE*_R,_ *SE*_A_) when the EM waves propagate along the aligned SWCNTs, the P-*SE*_R_ and P-*SE*_T_ (~ 51 dB) are slightly smaller because of the comparatively lower conductivity in the perpendicular direction (Fig. 3b). Even so, these *SE*_T_ values place our ultrathin SWCNT films among the best EMI shielding materials according to previously reported values [6, 8]. For example, the random network SWCNT buckypaper (previously discussed and having a lower electrical conductivity) can only reach EMI *SE* values of 28–45 dB even at much higher thicknesses of 18–60 µm, respectively (Fig. 3c). Furthermore, MWCNT assembled buckypapers with a conductivity of ~ 2,000 S m^−1^ show much lower EMI *SE* values. Indeed, the *SE* of the 120 µm thick buckypapers reaches only 14–20 dB, which can be attributed to the lower inherent conductivity of MWCNTs. Similarly, while graphene film is one of the most promising EMI shielding materials because of the high intrinsic conductivity of graphene, the conductivity of the most commonly used reduced graphene oxide (RGO) film is still limited because of the inevitable structural deterioration of graphene that occurs during the reduction process [21, 31]. Prepared RGO films with a conductivity of ~ 7,000 S m^−1^ and a density of ~ 2 g cm^−3^ reach an EMI *SE* value of ~ 22 dB at a thickness of 24 µm; thus, RGO films are vastly outperformed by the aligned SWCNT films (Fig. 3c). Moreover, the novel printing process can easily control the thickness of the produced freestanding SWCNT films, resulting in tunable EMI *SE* values because of the predetermined amount of SWCNTs interacting with the incident EM waves. The EMI *SE* reaches ~ 39 and ~ 90 dB at thicknesses of 1.5 and 24 µm, respectively (Fig. 3d). Note that higher thicknesses can be obtained for the SWCNT films (Fig. S1) and the EMI *SE* can reach above 100 dB, surpassing the upper limit for the instrument used in this experiment. In summary, the excellent EMI *SE* of the ultrathin aligned SWCNT films emerges from the film’s outstanding conductivity that itself emerges from the highly conductive SWCNTs once the bundles are properly aligned.

Other key factors that contribute to EMI shielding can be ascribed to the aligned cellular structure of the lightweight SWCNT film, which has a density of around 0.6 g cm^−3^ and a porosity of ~ 60%. The cellular structure offers increased surfaces for the multi-reflections of incident EM waves, resulting in increased opportunities for interactions between the wave and SWCNTs [2, 8, 14]. Moreover, the aligned SWCNT pattern in the films naturally leads to aligned pore channels in the incident EM waves’ propagation direction, which has been proven to be instrumental in enhancing the multi-reflections [54, 55]. This emergent behavior combines with the intrinsically high microwave absorption capability derived from high permittivity and abundant charge carriers of the SWCNTs to achieve the high *SE*_T_ (*SE*_T_ = *SE*_A_ + *SE*_R_) values observed [27, 56]. To further understand the influences of the aligned cellular structure on the EMI shielding performance, the experimental and theoretical EMI *SE* values were compared for SWCNT films of varied thicknesses (Figs. 3e and S11). For a film with a thickness of 8 μm, a consistent conductivity (242,988 S m^−1^) for the SWCNT film was used to calculate the theoretical *SE* (details included in the supplemental documentation) of a homogenous solid shield, yielding a *SE* of ~ 51.4 dB in the X-band frequency range. The lower theoretical *SE* compared to the experimental value is attributed to the contributions from multi-reflection and scattering of the incident EM waves within the aligned porous structure of the SWCNT films not considered in the theoretical model. Clearly, the experimental *SE* is close to the theoretical *SE* at low thicknesses but is higher than predicted value as sample thickness increases). This is a result of the increased pores in thicker samples that promote penetration and absorption of the incident EM waves [8, 14], enhancing experimental EMI *SE* values for the SWCNT films. It is the synergistic combination of the inherently excellent conductivity of SWCNTs, the aligned pattern, and the inherent cellular structure, that yields the ultrahigh EMI shielding performance at the low thicknesses and densities observed in our SWCNT films.

In addition, we have measured the SE in various frequency ranges, including 8.2–12.4 GHz (X-band), 12.4–18 GHz (Ku band), 33–50 GHz (Q-band), and 100–400 GHz (Terahertz band) for our SWCNT film (Fig. 3f). Our 8 µm thick SWCNT films can still reach an excellent SE value of more than 54 dB at a qualified bandwidth of around 400 GHz, far exceeding that of other reported shielding materials [32, 34, 57]. Furthermore, the SWCNT films maintain this excellent EMI *SE* even after a 10,000 cycle bending or rolling treatment (Fig. 3g), demonstrating excellent shielding stability and durability, and proving their application potential in practical EMI shielding applications, including applications involving an ultra-broadband frequency range.

Our films not only demonstrate robust shielding and conductivity performance when subjected to mechanical deformation, but can maintain excellent EMI shielding reliability during exposure to acids, alkalis, and solvents or high-/low-temperature environments. We have immersed separate SWCNT films for 15 days in concentrated hydrochloric acid (37%), sodium hydroxide solution (1 M), and acetone (Fig. S12). After these treatments, each SWCNT film still maintains good structural integrity and nearly identical EMI *SE* values compared to those measured before treatment (Fig. 3h). In contrast, traditional metal shields or metal nanomaterial-based counterparts cannot claim such results [6, 10, 27, 54]. In addition to maintaining excellent mechanical flexibility even while soaked in liquid nitrogen (−196 °C) (Fig. S13, Video S3), the SWCNT films exhibit identical EMI SE values after storage at a cryogenic temperature for 10 days (Fig. 3i). Similarly, even though the SWCNT films were exposed to 400 °C in atmosphere for 10 days, the EMI *SE* values were only slightly affected, demonstrating good thermal stability across a very broad range (Fig. S14). This is consistent with the behavior that the aligned SWCNT films have stable storage and loss modulus over a wide range of temperatures (Fig. S15), which further reveals the film’s superiority over most of the commonly used polymer-based shielding materials [21, 27]. The stability and reliability of our SWCNT film in a wide variety of harsh environments significantly add to its capacity as principal material for next-generation electronics.

**Fig. 4:**
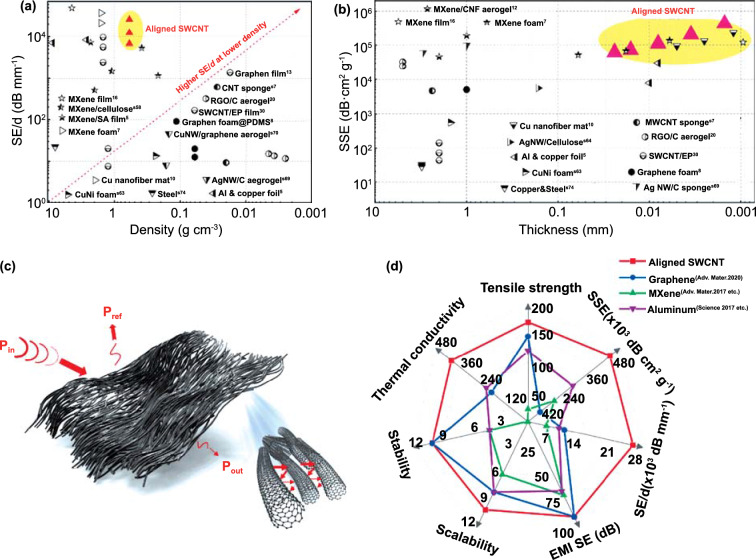
**a** Shielding mechanism of the aligned SWCNT films. Comparison of the aligned SWCNT shielding performance with other shielding materials: **b** SE/*d* values of the materials with various densities and **c** SSE values of the materials with various thicknesses. **d** Radar plots of the aligned SWCNT films with other shielding materials including some representative graphene [17], MXene [6, 8], and metal [6]-based materials

As discussed before, the inherently excellent conductivity of SWCNTs, the aligned pattern, and the inherent cellular structure have synergistically contributed to the excellent EMI SE of the aligned SWCNT films at a low density and ultralow thickness (Fig. 4a). Generally, improving the SE of the EMI shielding architectures while maintaining or decreasing thicknesses and densities is an immense challenge for already lightweight and high-performing EMI shields [6, 10, 14, 17, 20, 54]. To more directly evaluate the shielding performances of reported materials competing in this space, EMI SE divided by the thickness (*SE*/*d*) values of these shielding materials, along with their densities, are compared (Fig. 4b) with further details tabulated (Table S2). Our aligned SWCNT films’ thicknesses appear among the lowest values for the freestanding shielding architectures reported, while simultaneously satisfying industry standard SE requirements [2, 6, 14]. With film thicknesses from 1.5 to 24 µm, and a density of ~ 0.6 g cm^−3^, the *SE*/*d* of the SWCNT films ranges from 3 750 to 25 693 dB mm^−1^, outperforming that of most other shielding materials. With the additional consideration of the low density of our freestanding aligned SWCNT films, the *SSE* can reach an unprecedented value of up to 428,222 dB·cm^2^ g^−1^, significantly surpassing that of the other shielding materials, including carbon-based solid and porous materials, metal‐based solid materials and foams, and MXene-based shields (Fig. 4c, Table S2). Perhaps more impressive, with an exceptionally high EMI *SE* value of around 90 dB, the 24 µm thick, aligned SWCNT film could simultaneously reach an ultrahigh *SSE* value of 62,500 dB cm^2^ g^−1^. In sharp contrast, typical metal shields such as solid copper or stainless steel (~ 32 dB cm^2^ g^−1^) and most commonly used polymer nanocomposites have SSE values of a few tens to hundreds of dB cm^2^ g^−1^—three to four orders of magnitude lower than the aligned SWCNT films (Table S2) [27, 58]. The SWCNT films outperform traditional shields so extremely due to the emergent mechanisms resulting from the interplay of highly conductive aligned SWCNTs and the film’s cellular structure. When the film’s other advantages, including high thermal conductivity, reliability in extreme environments, facile production, and scalable fabrication are considered in totality with an outstanding EMI shielding performance (Fig. 4d, Table S1), our lightweight, ultrathin, flexible, strong, and waterproof SWCNT films offer tremendous promise for applications in aircraft, spacecraft, defense systems, portable electronics, smart wearable devices, and much more beyond the limits of present imagination.

## Conclusions

We premiere a printing fabrication method for a novel, lightweight, flexible, and freestanding aligned SWCNT film with high mechanical strength, good hydrophobicity and waterproof ability, excellent thermal and electrical conductivities, and a remarkable EMI shielding performance. Furthermore, this excellent EMI shielding performance remains robust when subjected to mechanical deformation, corrosive chemicals (acid, alkali, or organic solvent), and high-/low-temperature treatments. The synergy of the SWCNTs’ high intrinsic conductivity, unique cellular structure, and the film’s aligned pattern gives rise to an EMI *SE* of ~ 39–90 dB at thicknesses of merely 1.5–24 µm with a density of 0.6 g cm^−3^. The SE/*d* and SSE of the freestanding SWCNT films are up to 25 693 dB mm^−1^ and 428 222 dB·cm^2^ g^−1^, respectively, significantly surpassing those of previously reported shielding materials. In addition, the aligned SWCNT films display high EMI *SE* in an ultra-broadband frequency range of around 400 GHz. Finally, and perhaps most promising, the efficient, facile, environmentally friendly, and easily scalable fabrication method used to print the SWCNT films for this study can easily integrate functional additives such as magnetic nanomaterials, metal nanomaterials, or polymers into future films, which can further broaden the film’s application potentials within next-generation electronics.

## Supplementary Information

Below is the link to the electronic supplementary material.

Supplementary file1 (PDF 1883 kb)

Supplementary file2 (MP4 12960 kb)

Supplementary file3 (MP4 964 kb)

Supplementary file4 (MP4 1991 kb)
