# Supplementary material for: Printable Aligned Single-Walled Carbon Nanotube Film with Outstanding Thermal Conductivity and Electromagnetic Interference Shielding Performance
Source: Nanomicro Lett. 2022 Sep 1;14:179. doi: 10.1007/s40820-022-00883-9 (PMC9437195; doi:10.1007/s40820-022-00883-9)
Supplement: Supplementary file 1 — Supplementary file1 (PDF 1883 kb) [file 40820_2022_883_MOESM1_ESM.pdf]

Supporting Information for

Printable Aligned Single-Walled Carbon Nanotube Film with Outstanding Thermal Conductivity and Electromagnetic Interference Shielding Performance

Zhihui Zeng[1, §], Gang Wang[2, §, *], Brendan F. Wolan[2], Na Wu[3], Changxian Wang[4], Shanyu Zhao[5], Shengying Yue[6], Bin Li[1], Weidong He[5], Jiurong Liu[1, *], Joseph W. Lyding[2, *]

[1]Key Laboratory for Liquid-Solid Structural Evolution and Processing of Materials, Ministry of Education and School of Materials Science and Engineering, Shandong University, Jinan 250061, People's Republic of China

[2]Beckman Institute for Advanced Science and Technology, Department of Electrical and Computer Engineering, University of Illinois at Urbana-Champaign, Urbana, Illinois 61801, United States

[3]Department of Chemistry, Swiss Federal Institute of Technology in Zurich (ETH Zürich), 8092 Zürich, Switzerland

[4]School of Materials Science and Engineering, Nanyang Technological University, 50 Nanyang Avenue, Singapore 639798, Singapore

[5]Empa, Swiss Federal Laboratories for Materials Science and Technology, Überland Strasse 129, 8600 Dübendorf, Switzerland

[6]Institute for advanced technology, Shangdong University, Jinan 250061, People's Republic of China

§ Zhihui Zeng and Gang Wang contributed equally to this work.

*Corresponding authors. E-mail: wangg@gmail.com (Gang Wang), jrliu@sdu.edu.cn (Jiurong Liu), lyding@illinois.edu (Joseph W. Lyding)

S1 Supplementary Materials and Methods

S1.1 Fabrication of Random Network SWCNT and MWCNT Buckypapers, and the Reduced Graphene Oxide (RGO) Films

The random network SWCNT Buckypapers were prepared by filtrating the as-prepared SWCNT dispersion with a cellulose membrane filter (0.22 μm pore size). DI water was employed to remove the surfactant. The resulting dried films were peeled off from the substrate cellulose membranes. In the same procedure, the different aspect-ratios MWCNT assembled Buckypapers were also prepared based on the commercial MWCNT aqueous dispersions (supplied by Chengdu Institute of Organic Chemistry, Chinese Academy of Sciences): long MWCNT (TNM2, diameter around 8–15 nm and length around 50 μm) and short MWCNT (TNM8, average diameter around 50 nm and length 10–20 μm). The graphene films were prepared by the same vacuum filtration approach to the GO dispersion (prepared by a modified Hummer's method as reported in our previous work [23]) followed by a further reduction treatment by utilizing hydroiodic acid vapor as a reducing agent.

S1.2 Theoretical Simulation of SWCNT's Thermal Conductivity

To indicate the thermal conductivity (κ_{ph}) theoretically, we calculated the lattice thermal conductivity by solving the phonon Boltzmann Transport Equation (BTE) with the first-principles simulations. The models we adopted are armchair SWCNTs (8,8) and (12,12), which

are corresponding the diameter 1.085 and 1.6272 nm. Our simulating results of the lattice thermal conductivities along the axial direction at 300K are shown in Figure S5. κ_{ph} of the SWCNT (8,8) is 3580 W m^{-1}K^{-1}, and κ_{ph} of the SWCNT (12,12) is 3102 W m^{-1}K^{-1}. Our simulation results are consistent with other theoretical and experimental work [S1-S7]. Here, we only considered the κ_{ph} along the axial direction along the SWCNTs.

Here is the details of the theory and simulating process. First, by solving the BTE, we have the following equation:

$$\kappa_{ph} = \sum_p \sum_q C_{ph} \cdot v_g^2 \cdot \tau \qquad \text{(S1)}$$

where p and q donate the phonon branch and wavevector respectively, C_{ph} is the specific heat capacity of phonon,v_g is the phonon group velocity along the axial direction of SWCNTs and τ is the phonon lifetime. To obtain the key parameters v_g and τ, we calculated the second and third order interatomic force constants (IFCs) based on the density functional theory (DFT) method. We performed all the first-principles calculations with the Vienna Ab-initio Simulation Package (VASP) [S8, S9], and chosen the Perdew-Burke-Ernzerhof (PBE) of the generalized gradient approximation (GGA) as the exchange correlation functional [S10]. We used the projector augmented wave (PAW) potentials to describe the core ($1s^2$) and valence electrons ($2s^2$ and $2p^2$) of carbon element. The kinetic energy cutoff of the wave functions was set as 500eV, which is enough for the hard-core carbon element. In the momentum space of electrons, the k-mesh 1×1×20 was used to sample the Brillouin Zone (BZ) including Γ point by Monkhorst-method. To hinder the self-interactions among the cylinders arising from the employed periodic boundary condition, we set a vacuum layer 10 Å among the neighbor unit cells. All the structures were fully optimized with the Hellmann-Feynman force tolerance $0.001\ eV/\text{Å}$. For the second and third order IFCs calculations, the supercell 1×1×6 was constructed, the convergence of length was examined. The second order harmonic IFCs were obtained under the linear response framework by using the finite displacement method as implemented in the PHONOPY package [S11]. The phonon dispersions can also be obtained from PHONOPY package, which is shown in Fig. S1. In the calculations of third order IFCs, the atomic force interaction cutoff was taken into account up to forth nearest neighbors. With the third order IFCs, we iteratively solved the phonon BTE by using ShengBTE code developed by Li et al. [S12]. The momentum space of phonon was sampled with 1×1×100 grid in first BZ. Then we have the phonon lifetime τ, and combining with the Eq. (1) we calculated the lattice thermal conductivity κ_{ph} finally. The convergence of the sampling grid in momentum space for phonon was examined [S12]. To convert the ShengBTE results into the experimental values, we used the cross-sectional area $S = \pi d h$, for the SWCNTs [S13], where d is the diameter and $h = 3.4$ Å (the interactive length which is the van der Waals force radius of carbon atoms).

S1.3 Thermal Conductivity Measurement

Thermal conductivity of as-prepared freestanding SWCNT film was measured through our home-made vacuum thermal test apparatus (Fig. S6a). The as-prepared SWCNT films were cut into strips with width of around 5 mm and suspended in a vacuum chamber between two isolated stages, which were connected to alumel-chromel thermocouples. The two stages were named as floating and fixed stages, respectively. The floating stage was placed on Teflon tubes and did not make surface contact with anything other than the test sample. The fixed stage was connected to a power supply and could be warmed up by Joule heating. After loading sample, the apparatus was pumped down with a mechanical pump to reduce the chances of causing heat dissipation through convection. The thermal measurement was triggered by turning on the power supply attached to a heater on the fixed stage. The temperatures of the two stages were then recorded through the stage thermocouples every 60 seconds for thirty minutes, and the

power supply was shut off for the final ten minutes. The thermal conductivity of the sample was calculated in between every two measured points using the following equations and then averaged together (Fig. S6b).

$$\frac{\Delta T}{R} = mc_p\frac{\partial T}{\partial t} + \varphi\Delta T_{leakage} \quad (S2)$$

$$\Delta T \equiv T_{fixed} - T_{float} \quad (S3)$$

$$\Delta T_{leakage} \equiv T_{float} - T_{ambient} \quad (S4)$$

$$R_{sample} = \frac{L}{kA} \quad (S5)$$

Here, m is the mass of copper and stainless steel of the floating stage, c_p is the heat capacity of the metals of the floating stage, T_{fixed} is the temperature of fixed stage measured during test, T_{float} is the temperature of floating stage measured during test, R_{sample} is the absolute thermal resistance of the sample, L is the thickness of the sample, which can be measured before test, A is the cross-sectional area perpendicular to the path of heat flow, k is the thermal conductivity of the sample.

It can be found that we introduced a correction term $\varphi\Delta T_{leakage}$ compared to the standard heat equation. It is because the heat leaking from the floating stage, which should be proportional to the temperature difference between the floating stage and room temperature ambient, is non-negligible. φ is an empirical constant that can be obtained through adjusted it until the thermal conductivity is close to a constant. For example, as shown in Figure S6c, the measured thermal conductivity of pure copper without leakage correction is displayed as blue squares, which shows an unreasonably non-constant feature. After introducing leakage correction, measured thermal conductivity of copper (red dots in Fig. S6c) became constant and close to its theoretical value. We also measured some pure metals (copper, aluminum and silver) and found their measured thermal conductivity values were consistent with their theoretical values (Fig. S6d).

S1.4 EMI Shielding Performance Test

The EMI shielding tests of the samples were carried out with the waveguide method by a vector network analyzer (VNA, Agilent 8517A). The tested samples were cut with size of 22.86×10.16 mm^2 (length × width) for the X-band frequency range of 8.2–12.4 GHz, 15.8×7.9 mm^2 (length × width) for the Ku-band frequency range of 12.4–18 GHz, and 5.68×2.84 mm^2 (length × width) for the Q-band frequency range of 33–50 GHz. Herein, the thin samples were fixed between two 1 mm-thick PC substrates with negligible EMI SE. In the Terahertz frequency range of 100–400 GHz, shielding performance was evaluated using terahertz time domain spectroscopy (Topical Teraflash). More than five samples for each component were tested. Unless specifically mentioned, the electric field direction of the incident EM waves was parallel to the aligned SWCNTs for the SWCNT films. The obtained S-parameters of each sample were used to calculate the EMI SE as follows:

$$SE_T = -10Lg\,(|S_{12}|^2) = -10Lg\,(|S_{21}|^2) \quad (S6)$$

$$SE_R = -10Lg\,(1-|S_{11}|^2) \quad (S7)$$

$$SE_A = -10Lg\,(|S_{12}|^2/\,(1-|S_{11}|^2)) = SE_T - SE_R \quad (S8)$$

Where $|S_{ij}|^2$ is the power transferred from port i to port j.

S1.5 Theoretical Calculation of EMI Shielding Performance

The complex transmission coefficient (T) of a homogeneous shield can be calculated by a Transfer Matrix Method. The continuity of the tangential parts of both electric and magnetic fields of a time harmonic ($e^{j\omega t}$) plane wave at the incident face of shields generate the boundary conditions:

$$\begin{cases} A_{0i}e^{-ik_0z_0} + B_{0r}e^{ik_0z_0} = A_1e^{-ik_1z_0} + B_1e^{ik_1z_0} \\ Y_0(A_{0i}e^{-ik_0z_0} - B_{0r}e^{ik_0z_0}) = Y_1(A_1e^{-ik_1z_0} - B_1e^{ik_1z_0}) \end{cases}, \quad \text{(S9)}$$

where A and B are the coefficients of forward-travelling and backward-travelling waves, respectively, $k = \sqrt{\mu\varepsilon}$ is the wave number, $Y = \sqrt{\varepsilon/\mu}$ is the admittance of shielding materials, μ and ε are the complex permeability and permittivity of shielding materials, the subscripts 0 and 1 are variables relating to the air and the shielding materials, respectively. Since our shielding architectures are nonmagnetic, μ equals to 1. The complex permittivity (ε) consist of the real part (ε') and imaginary part (ε'')as following:

$$\varepsilon = \varepsilon' - j\varepsilon'' = \varepsilon'(1 - j\frac{\sigma}{w\varepsilon'}). \quad \text{(S10)}$$

where w is the angular frequency and σ is the conductivity. Herein, for a conductivity-caused EMI shielding calculation of the homogenous shields, the real part (ε') is equal to ε_0.

Thus, the boundary condition gives as follows at the wave emergent face of the shielding materials:

$$\begin{cases} A_1e^{-ik_1z_1} + B_1e^{ik_1z_1} = A_{0t}e^{-ik_0z_1} \\ Y_1(A_1e^{-ik_1z_1} - B_1e^{-ik_1z_1}) = Y_0A_{0t}e^{-ik_0z_1} \end{cases}. \quad \text{(S11)}$$

The T of the shielding materials are calculated as follows:

$$T = \frac{A_{0t}}{A_{0i}} \quad \text{(S12)}$$

Finally, the SE_T of the shields in dB are calculated:

$$SE_T = 10\log\frac{1}{T^2} \quad \text{(S13)}$$

S2 Supplementary Videos

Video S1 The printing process for preparing the SWNCT film

Video S2 The SWCNT film after immersion in water for 15 days and sonication treatment

Video S3 The SWCNT film immersed in liquid nitrogen

S3 Supplementary Figures and Tables

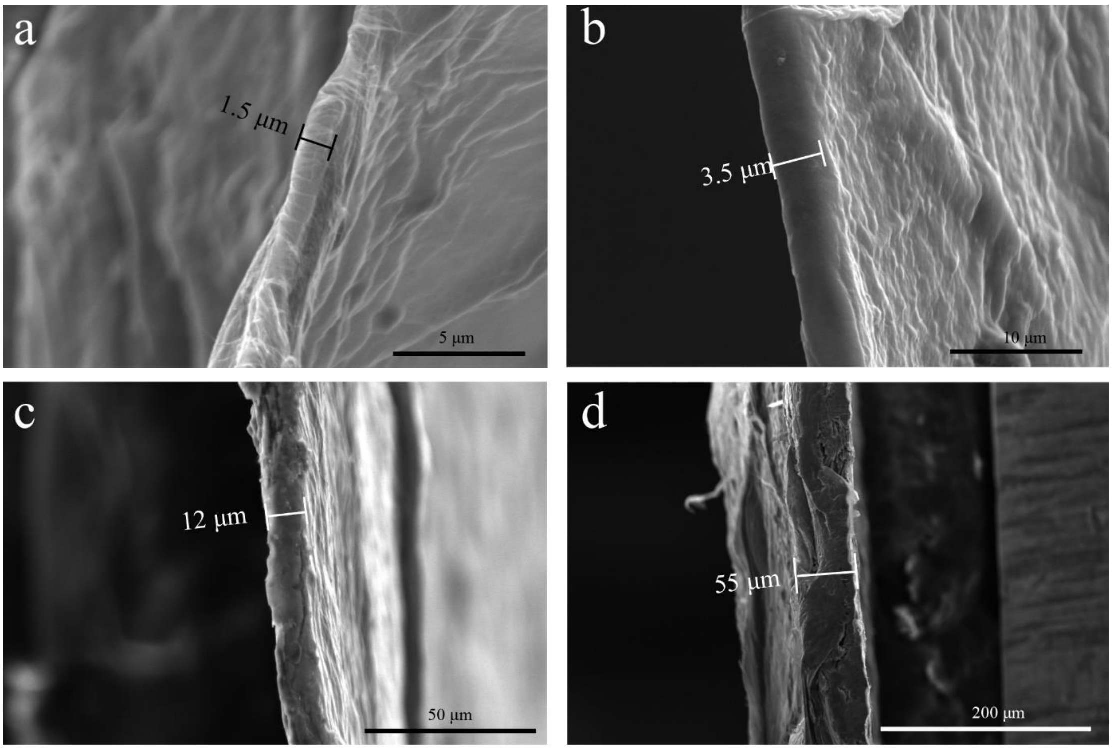

Fig. S1 SEM images of as-prepared freestanding SWCNT films with various thickness: (**a**) 1.5 μm, (**b**) 3.5 μm, (**c**) 12 μm, and (**d**) 55 μm

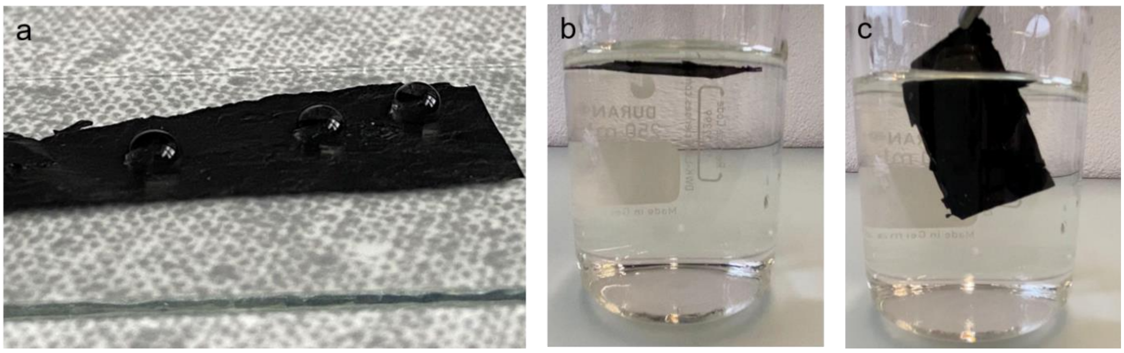

Fig. S2 Optical images of the hydrophobic and waterproof aligned SWCNT films (**a**) with water drops on the surface, after (**b**) immersion in water for 15 days and (**c**) a further ultrasonic treatment of 10 min

Calculation of porosity of the aligned SWCNT films. The porosity of the SWCNT films is calculated by ($1 - \rho / \rho_0$), where ρ and ρ_0 are the density of the SWCNTs and SWCNT films, respectively. The apparent densities of the SWCNT films are obtained by the weighing method and combined with the density of SWCNTs (~1.5 g cm^{-3}). Herein, the porosity of the aligned SWCNT films with an apparent density of ~0.6 g cm^{-3} can be calculated with a value of 60%.

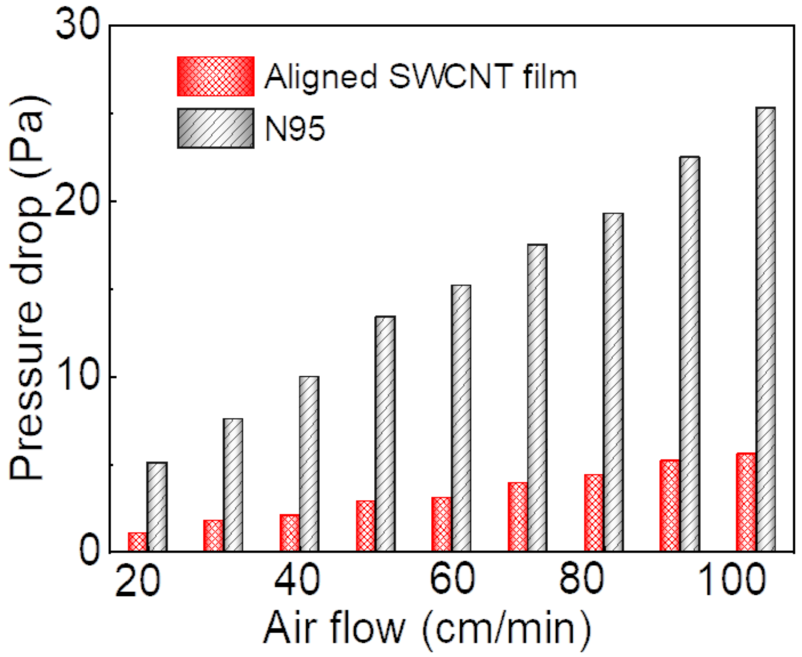

Fig. S3 Pressure drop of the aligned SWCNT films in comparison to the commercial N95 masks. The much lower pressure drop of the SWCNT films shows the good air permeability

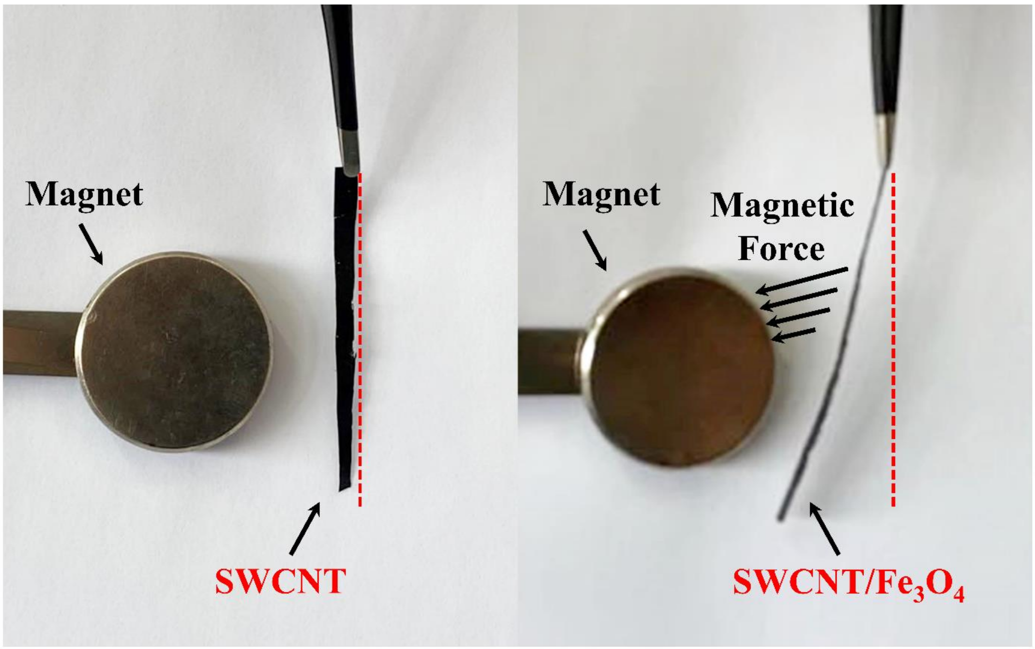

Fig. S4 Optical images of the printed SWCNT film (left) and the SWCNT film doped with magnetic Fe_3O_4 (right) attracted by a magnet, showing the capability of integrating other functional materials

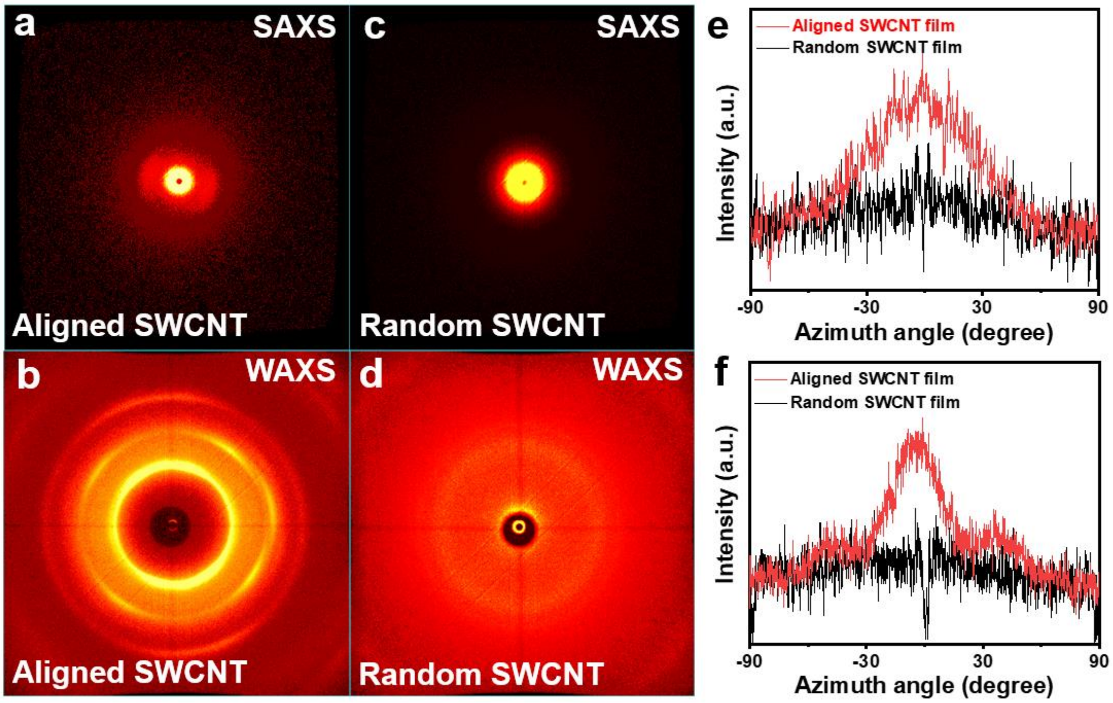

Fig. S5 2D SAXS/WAXS patterns and azimuthal-integrated intensity distribution curves of SWCNT films. (**a**) SAXS patterns and (**b**) WAXS patterns of aligned SWCNT films; (**c**) SAXS patterns and (**d**) WAXS patterns of random SWCNT films; (**e**) azimuthal-integrated intensity distribution curves from SAXS and (**f**) WAXS patterns of aligned SWCNT and random SWCNT films

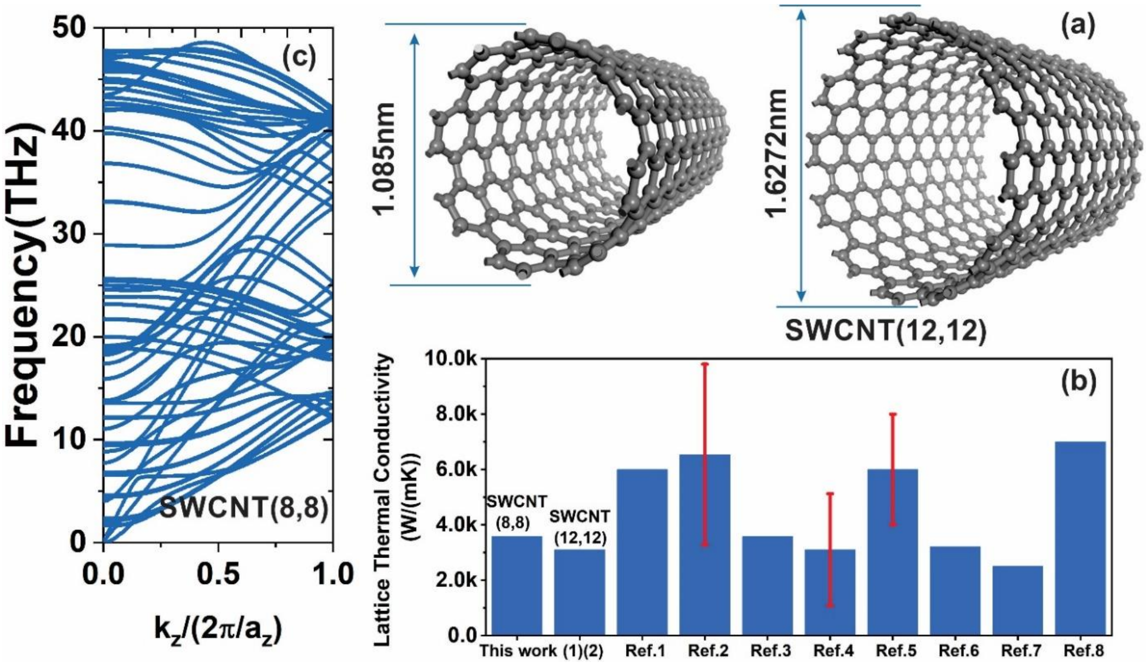

Fig. S6 (**a**) The simulating models of SWCNTs with diameters of 1.085 nm (8,8) and 1.6272nm (12,12). (**b**) The lattice thermal conductivities of SWCNTs from our simulations and references. (**c**) The phonon dispersion of the SWCNT (8,8) with diameter of 1.085 nm

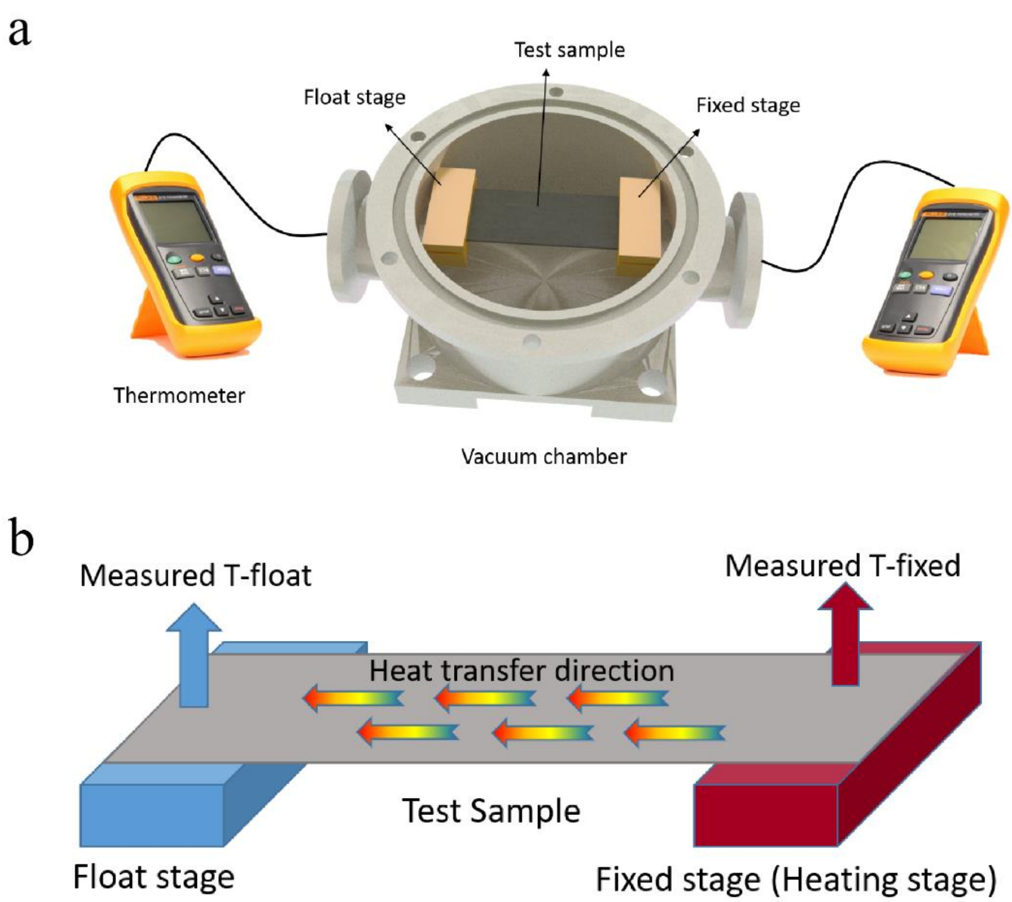

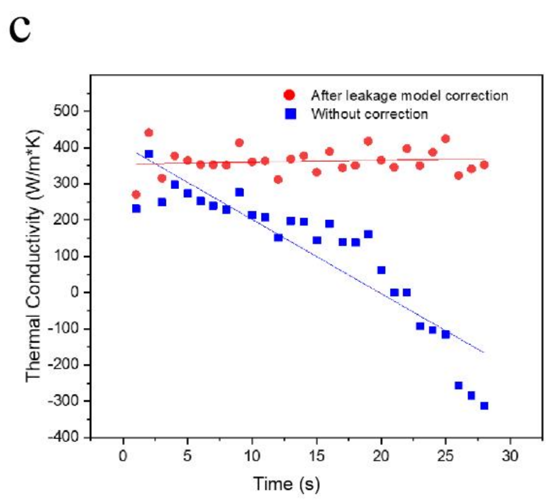

d

	Experiment (W/m*K)	Theory (W/m*K)
Copper	377	401
Aluminum	235	237
Silver	395	429

Fig. S7 (**a**) Schematic of the thermal conductivity measurement apparatus. (**b**) Schematic of the thermal conductivity measurement. (**c**) Comparison of thermal conductivity results obtained with and without leakage correction. (**d**) Comparison of measured thermal conductivity results of some pure metal metals (copper, aluminum and silver) and their theoretical values

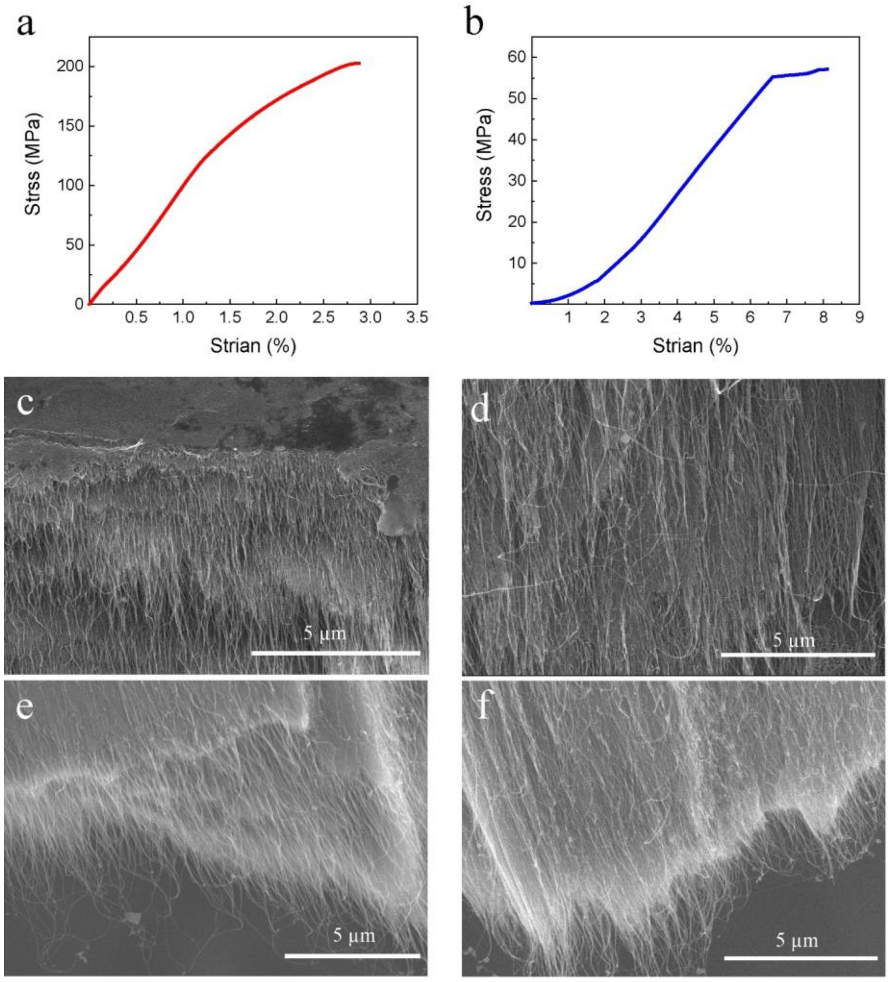

Fig. S8 Typical stress-strain curves of freestanding SWCNT films in (**a**) parallel and (**b**) perpendicular directions. (c-f) SEM images of the microstructures of the as-prepared SWCNT film's fracture surface measured from parallel direction

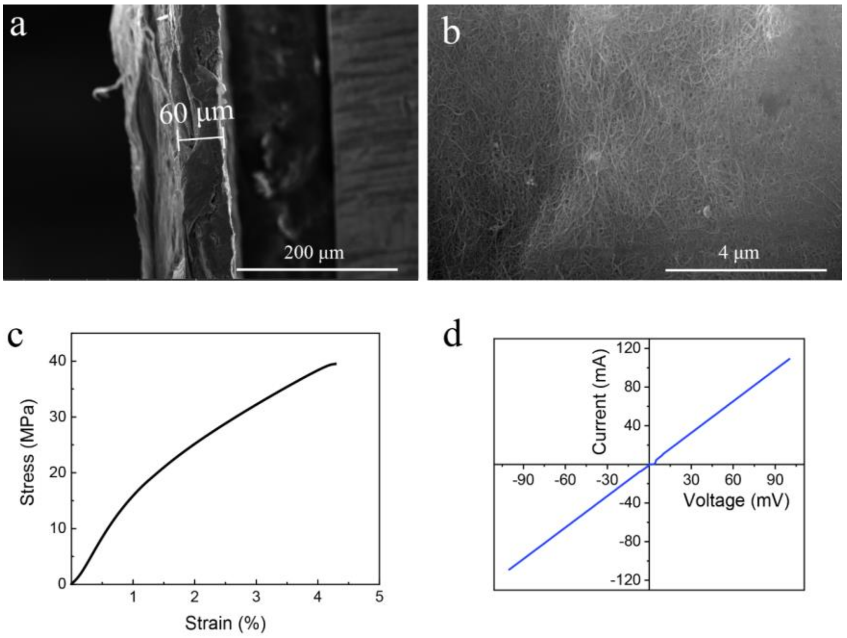

Fig. S9 Characterizations of random network SWCNT Buckypaper. (**a**) Side-view SEM image, showing a typical thickness of 60 µm. (**b**) In-plane SEM image of the Buckypaper, showing random network of SWCNTs. (**b**) Typical tensile stress-strain curve. (**d**) I-V curve

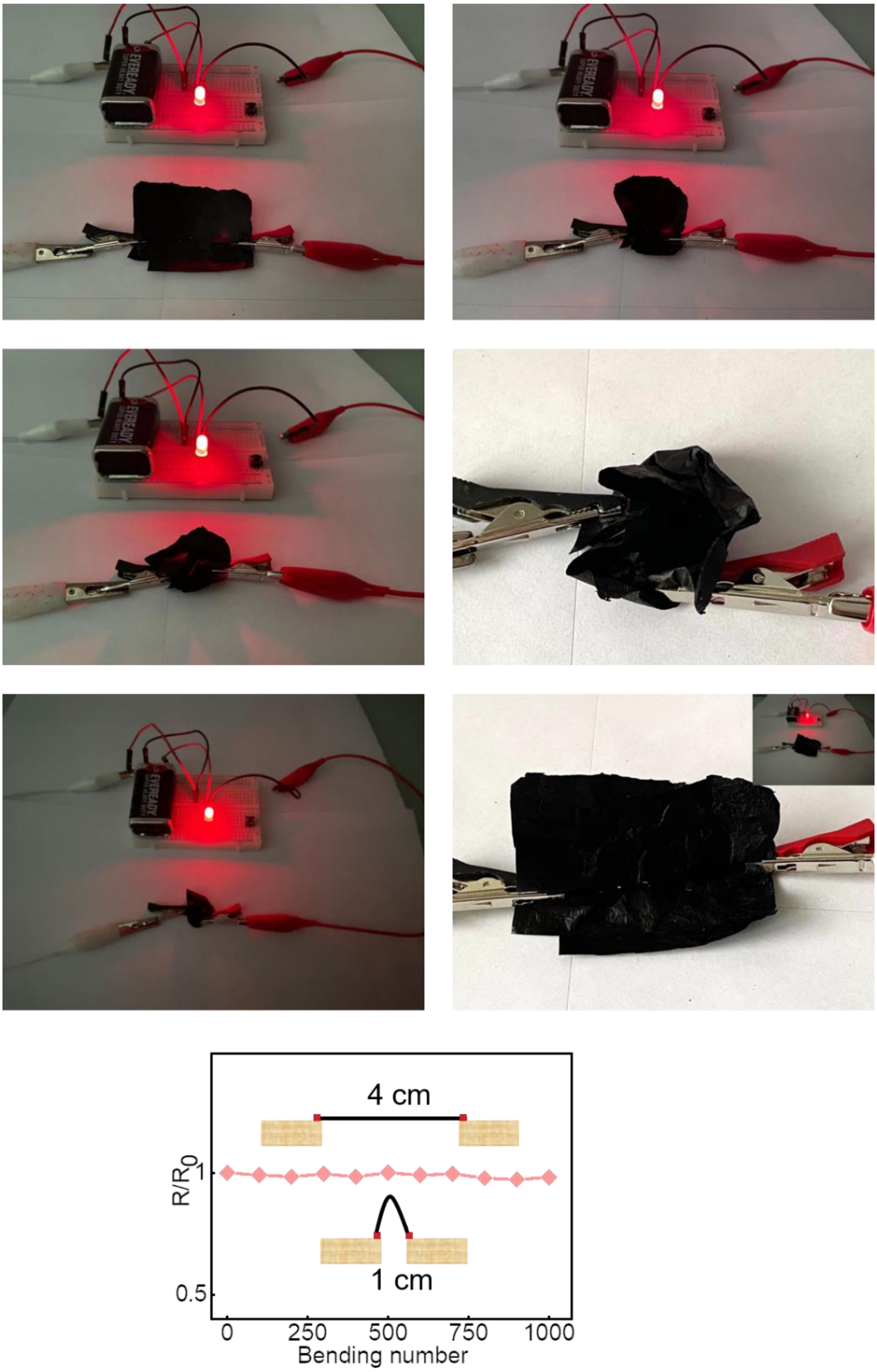

Fig. S10 Demonstrations of the aligned SWCNT film as a flexible electrical conductor before and after mechanical deformations including bending, twisting, and kneading, and ohmic resistance change of an SWCNT film during the 10000-cycle bending measurement. These show the stability and reliability of the SWCNT films for ultraflexible electronics. The cycle bending treatment was performed at a bending speed of 3 mm s^{-1} and a bending angle of around 30°

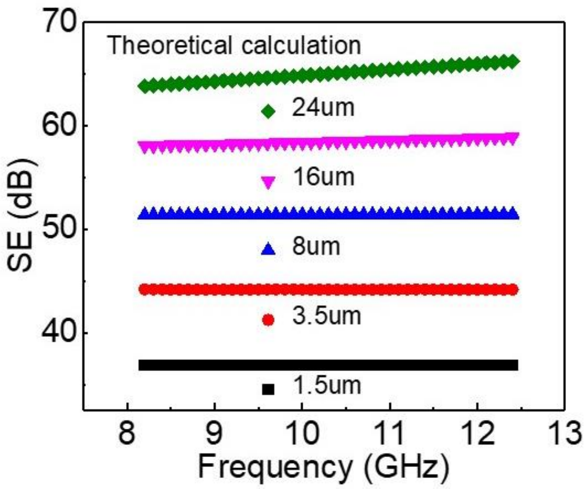

Fig. S11 Theoretically calculated EMI SE based on the conductivity of a homogeneous shield with various thicknesses

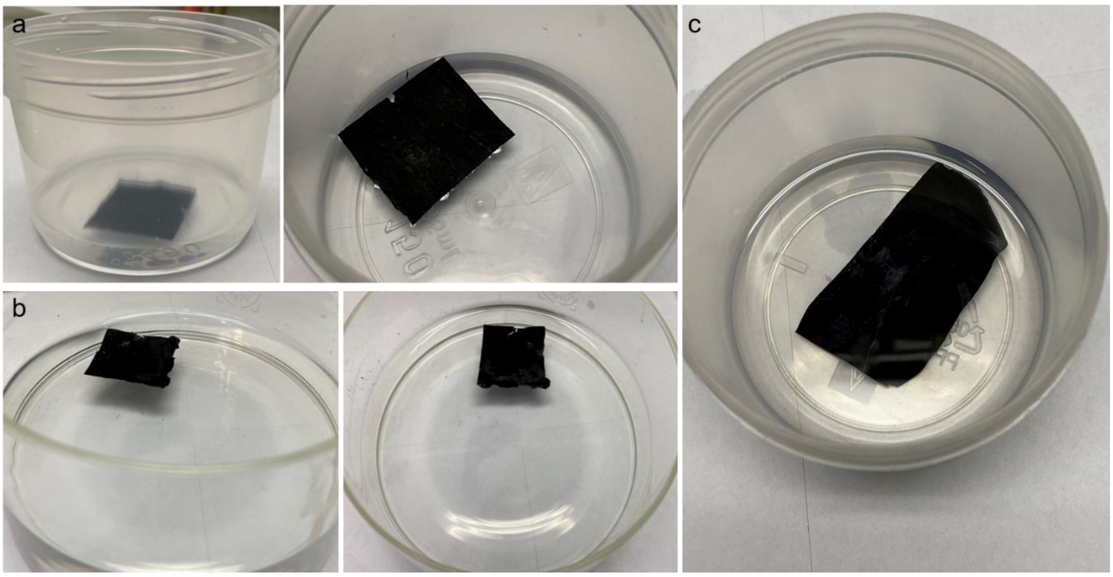

Fig. S12 Optical images of the aligned SWCNT films after immersion for 15 days in (**a**) concentrated hydrochloric acid (37%), (**b**) sodium hydroxide (1M), and (**c**) acetone

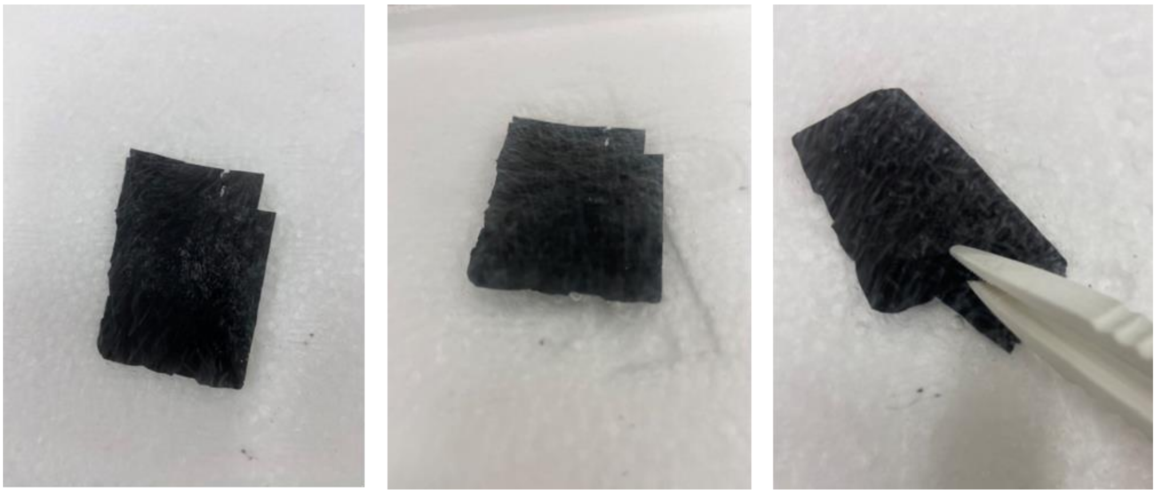

Fig. S13 Optical images of the aligned SWCNT films in liquid nitrogen (-196 ℃), showing the stability and mechanical flexibility of the aligned SWCNT films under extremely low temperature conditions

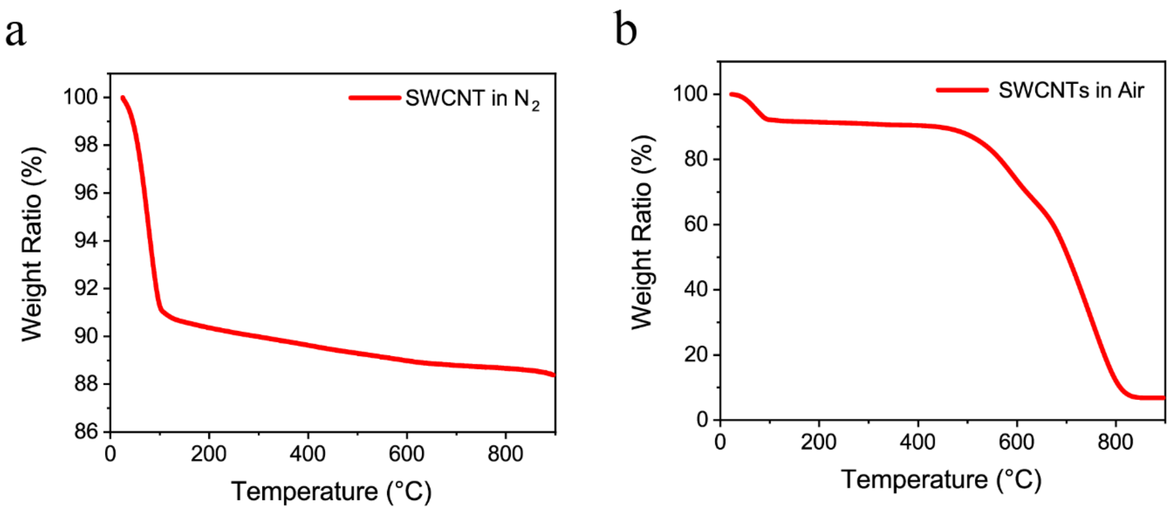

Fig. S14 Thermal gravimetric analysis of SWCNTs in (**a**) nitrogen, and (**b**) air from room temperature to 900 °C

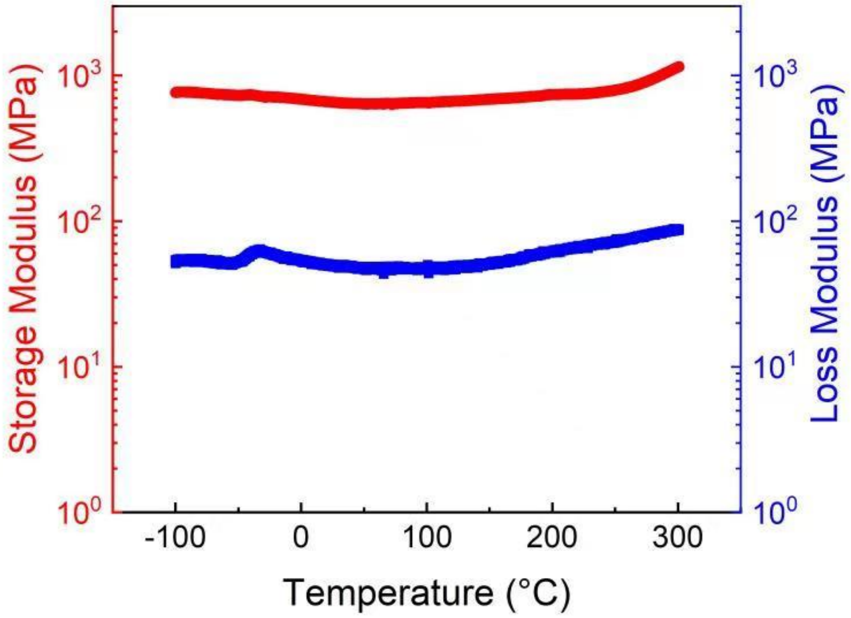

Fig. S15 Storage and loss modulus of the aligned SWCNT films in a wide range of temperature, showing the stability of the SWCNTs in low/high temperature conditions

Table S1 Comprehensive properties comparison of various EMI shielding materials

Materials	Tensile strength (MPa)	Thermal conductivity (W $m^{-1}K^{-1}$)	Refs.
Aligned SWCNT film	169	398	This work
Graphene film	145	190	[S14]
Graphene/PI film	128	142	[S14]
Al-foil	120	217	[S15, S16]
$Ti_3C_2T_x$-MXene film	22	2.84	[S17, S18]
90wt%-$Ti_3C_2T_x$ MXene/SA	50	< 2.84	[S17, S18]
16wt%-rGO/PI	11.4	< 20	[S19, S20]

Table S2 EMI shielding performance of various shielding materials

Materials	EMI SE (dB)	Thickness (mm)	SE/d (dB mm^{-1})	SSE (dB·cm^2 g^{-1})
**Carbon-based porous and solid shields**				
Aligned Cellular SWCNT film (This work)	38.54<br>43.11<br>54.43<br>~90	0.0015<br>0.0035<br>0.008<br>0.024	25 693<br>12 317<br>6 804<br>3 750	428 222<br>205 286<br>113 395<br>62 500
Random network SWCNT buckypaepr (this work)	45	0.06	7500	25000
SWCNT/MWCNT buckypaper [S21]	65	0.13	500	6098
MWCNT buckyaper [S22]	43	0.13	330.8	5803
SWCNT nanopaper [S22]	57	~0.05	1140	12667
MWCNT nanopaper [S22]	30	~0.05	600	10909
MWCNT/PLA foam [S23]	23	2.5	9.2	308
MWCNT/PVDF foam [S24]	57	2	28.5	380
MWCNT/WPU foam [S25]	23.0<br>21.1	2.3<br>1	10<br>21.1	4991<br>5410
MWCNT/cellulose aerogel [S26]	20-35	2.5	8-14	1700-3776
CNT sponge [S27]	22	2.38	9.2	4622
Cellulose aerogel coated with MWCNT [S26]	35-40	2.5	1416	1864-2078
Graphene foam based PDMS foam [S28]	30	1	30	~5000
Graphene foam/CNT/PDMS [S29]	75	2	37.5	4165
Graphene-coated PU foam [S30]	19.9	20	~1	3320
Graphene foam coated with PEDOT:PSS [S31]	69.1	1.5	46.1	20837
Graphene based composite aerogel [S32]	37	3	12.3	1762
Sponged-supported RGO aerogel [S33]	24	12	2	1198
CNT/multi-layered graphene foam [S34]	~38	1.6	23.8	~40000
Graphene/cellulose-derived carbon foam [S35]	47.8	5.0	9.6	33780
Graphene/WPU [S36]	32	2	16	153
CNF/PS foam [S37]	19	/	/	/
CNT/PS foam [S38]	19	/	/	/
Graphene/PVDF foam [S39]	28	/	/	/
Graphene/PMMA foam [S40]	19	2.4	7.9	100
Graphene/PS foam [S41]	29	2.5	11.6	258
Graphene /PEI foam [S42]	9-12.8	2.3	3.9-5.6	135–192
Graphene/lignin-derived carbon aerogels [S43]	23.2<br>14.3	2<br>1	11.6<br>14.3	46400<br>57200
Graphene aerogel [S44]	22.3	2	11.15	24778
Carbon/Graphene foam [S45]	24	0.024	1000	13889
Graphene foam [S46]	25.2	0.3	84	14000
Graphene@Fe_3O_4/PEI foam [S46]	15-18	2.5	6-7.2	150–176
CF/PP foam [S47]	25	3.1	8.1	109
Stainless-steel fiber/PP foam [S48]	48	3.1	15.5	242
Phthalonitrile-based carbon	51.2	2	25.6	1707

foam [S49]				
Commercial carbon foam [S50]	40	2	20	1250
Carbon foam-CNT/carbon fiber foam [S51]	21	5.0	4.2	3370
SWCNT/cellulose film [S52]	~35	0.0315	1111.1	7678
MWCNT/cellulose film [S53]	~20	0.0308	649.4	4205
SWCNT(long)/epoxy [S53]	25	2	12.5	72
SWCNT(annealed)/epoxy [S53]	21	2	12	60
SWCNT(short)/epoxy [S53]	16	2	8	46
SWCNT/epoxy [S54]	15-49	2	7.5-24.5	43-141
SWCNT/PU [S55]	18	2	9	80
CF mat [S56]	23	0.06	383.3	/
Ni/CF mat [S56]	29	0.06	483.3	/
Fe_3O_4/CNF mat [S57]	68	0.7	97.1	/
CNF mat [S58]	81.1	4.6	17.6	804.3
	52.2	2.9	18	1361.6
MWCNT/PTT [S59]	22	2	11	/
MWCNT/PP [S60]	24	2.8	8.6	95
	35	1.0	35	/
CNF sponge/Epoxy [S61]	40	2	20	/
MWCNT/ABS [S62]	50	1.1	45.5	433
Carbon black (CB)/ABS [S62]	22	1.1	20	190
Carbon nanofiber (CNF)/ABS [S62]	35	1.1	31.8	/
MWCNT/WPU [S63]	24-50	0.05-0.32	480	3408
MWCNT/PC [S64]	25	1.85	13.5	
MWCNT/PS [S65]	60	2	30	285
Graphene/CNA [S66]	58.4	2.0	29.2	/
CB/EPDM [S67]	18	5.5	3.27	/
CVD graphene paper [S68]	62	0.05	1200	18300
Pristine graphene/PI film [S14]	43.8	0.01	4380	28627
	60.2	0.025	2408	16161
Pristine graphene film [S14]	38.1	0.004	9525	63926
	44.5	0.008	5563	37332
Flexible Graphite [S69]	110	0.2	550	500
**MXene-based porous and solid shields**				
MXene/CNF aerogel [S70]	74.56	2.0	37.3	46600
	28.41	1	28.4	189400
MXene ($Ti_3C_2T_x$) foam [S71]	32	0.006	5333.3	137000
	70	0.06	1166.7	53030
MXene aerogel [S72]	61.2	2	30.6	49520
MXene/CNT aerogel [S73]	104	42	3	2476
MXene-POSS-NH_2 aerogel [S74]	34.5	/	2	/
MXene/PVA aerogel [S75]	28	10.8	5	2586
MXene/SA film [S76]	57	0.008	7125	30830
MXene film [S76]	68	0.011	6182	25863
MXene/cellulose film [S77]	24	0.047	510.6	2647
	25	0.0167	1497	1326
MXene@PS solids [S78]	62	2	31	29.5
MXene/RGO-epoxy solids [S79]	56.4	2	28.2	/
MXene film (blade coated)[S80]	46.1	0.00094	49043	~120 000
**Metal-based porous and solid shields**				

Cu wrapped polymer nanofiber based porous membrane [S81]	44.71	0.0012	37258	232860
	53.2	0.0025	21280	133000
Ag wrapped polymer nanofiber based porous membrane [S81]	55.13	0.0025	22052	111939
Cu wrapped PVDF fiber based porous membranes [S81]	43.05	0.008	5381	28854
CuNi foam [S82]	15-25	1.5	10-16.7	420–690
CuNi-CNT foam [S82]	40-54.6	1.5	26.7-36.4	116–1580
Porous cellulose papers coated with Ag NWs [S83]	48.6	0.164	296	5584
AgNW/CNF [S84]	70.5	2	35.2	56854
	30.3	1	30.3	178235
AgNW/polymer Aerogels [S85]	36.4-72.5	2.3	15.8-31.5	11420-20522
Ag NWs/PI foam [S86]	17-23.5	5	3.2-4.7	2136 -1544
Ag NWs/WPU foam [S87]	20.0-64.0	2.3	8.7-27.8	10970-6184
Ag NW@C hybrid sponge [S88]	37.9	1	37.9	99214
	70.1	3	23.4	61169
Cu NWs aerogels [S89]	~17	9.46	1.8	/
Cu NW@ graphene aerogels [S89]	52.5	9.46	8.1	3921.8
Al foil [S75]	66	0.008	8250	30555
Cu foil [S75]	70	0.010	7000	7812
CA/AgNW/PU Film [S90]	31.2	/	/	/
PP/PDA/AgNPs/PDMS [S91]	71.2	1.5	47.5	1804.7
AgNW aerogel/PDMS	~60	4	15	/
PPy/PDA/AgNW [S92]	48.4	> 0.095	< 509.5	< 1819.5
Copper [S93]	90	3.1	29	32
Nickel [S93]	82	/		
Stainless steel [S93]	89	4	22.3	28
(2 µm) Ni fibers/PES [S93]	58	2.85	20.4	109
(20 µm) Ni fibers/PES [S93]	4	2.85	1.4	
Ni filaments/PES [S93]	~87	2.85	30.5	165
Aluminium flakes/PES [S94]	35-39	2.92	12-13.4	/
Ag NW/PANI [S95]	48	0.0133	3609	28872
Ag NW/epoxy [S96]	25.09	0.040	627	5018
Ag NP/epoxy [S97]	5.06	0.040	126.5	1012
Ag NW/PVA [S96]	30.1	0.040	752.5	6691
Ni-Co alloy nanoparticle-coated PAN-PU [S97]	68	0.18	377.7	640
Ag NW/graphene [S98]	26	0.03	867	/
Ag NW/PS [S99]	31.85	0.8	39.8	379
Cu NW/PS [S100]	35	0.21	166.7	158.7

/: unclear or uncalculated value; the numbers in the square brackets denote the numbers of references which are at the end of the supporting information.

Supplementary References

[S1] S. Berber, Y.K. Kwon, D. Tomanek, Unusually high thermal conductivity of carbon nanotubes. Phys. Rev. Lett. **84**, 4613 (2000). https://doi.org/10.1103/PhysRevLett.84.4613

[S2] C. Yu, L. Shi, Z. Yao, D. Lim, A. Majumdar, Thermal conductance and thermopower of an individual single-wall carbon nanotube. Nano Lett. **5**(9), 1842-1846 (2005). https://doi.org/10.1021/nl051044e

[S3] E. Pop, D. Mann, Q. Wang, K.E. Goodson, H.J. Dai, Thermal conductance of an individual single-wall carbon nanotube above room temperature. Nano Lett. **6**(1), 96-100 (2006). https://doi.org/10.1021/nl052145f

[S4] J. Hone, M. Whitney, C. Piskoti, A. Zettl, Thermal conductivity of single-walled carbon nanotubes. Phys. Rev. B **59**(4), R2514-R2516 (1999). https://doi.org/10.1103/PhysRevB.59.R2514

[S5] Z. Wang, D. Tang, X. Li, X. Zheng, W. Zhang et al., Length-dependent thermal conductivity of an individual single-wall carbon nanotube. Appl. Phys. Lett. **91**(12), 123119 (2007). https://doi.org/10.1063/1.2779850

[S6] L. Lindsay, D.A. Broido, N. Mingo, Diameter dependence of carbon nanotube thermal conductivity and extension to the graphene limit. Phys. Rev. B **82**(16), 161402 (2010). https://doi.org/10.1103/PhysRevB.82.161402

[S7] D. Donadio, G. Galli, Thermal conductivity of isolated and interacting carbon nanotubes: comparing results from molecular dynamics and the boltzmann transport equation. Phys. Rev. Lett. **99**(25), 255502 (2007). https://doi.org/10.1103/PhysRevLett.99.255502

[S8] G. Kresse, J. Furthmuller, Efficiency of ab-initio total energy calculations for metals and semiconductors using a plane-wave basis set. Comput. Mater. Sci. **6**(1), 15-50 (1996). https://doi.org/10.1016/0927-0256(96)00008-0

[S9] G. Kresse, J. Furthmuller, Efficient iterative schemes for ab initio total-energy calculations using a plane-wave basis set. Phys. Rev. B **54**(16), 11169-11186 (1996). https://doi.org/10.1103/PhysRevB.54.11169

[S10] J.P. Perdew, K. Burke, M. Ernzerhof, Generalized gradient approximation made simple. Phys. Rev. Lett. **77**(18), 3865-3868 (1996). https://doi.org/10.1103/PhysRevLett.77.3865

[S11] A. Togo, F. Oba, I. Tanaka, First-principles calculations of the ferroelastic transition between rutile-type and $CaCl_2$-type SiO_2 at high pressures. Phys. Rev. B **78**(13), 134106 (2008). https://doi.org/10.1103/PhysRevB.78.134106

[S12] W. Li, J. Carrete, N.A. Katcho, N. Mingo, ShengBTE: a solver of the boltzmann transport equation for phonons. Comput. Phys. Commun. **185**(6), 1747-1758 (2014). https://doi.org/10.1016/j.cpc.2014.02.015

[S13] A.M. Marconnet, M.A. Panzer, K.E. Goodson, Thermal conduction phenomena in carbon nanotubes and related nanostructured materials. Rev. Mod. Phys. **85**(3), 1295-1326 (2013). https://doi.org/10.1103/RevModPhys.85.1295

[S14] Q. Wei, S. Pei, X. Qian, H. Liu, Z. Liu et al., Superhigh electromagnetic interference shielding of ultrathin aligned pristine graphene nanosheets film. Adv. Mater. **32**(14), e1907411 (2020). https://doi.org/10.1002/adma.201907411

[S15] G. Marami, S.M. Saman, M.A.S. Sadigh, Enhanced mechanical properties of pure

aluminium: experimental investigation of effects of different parameters. J. Cent. South Univ. **25**(3), 561-569 (2018). https://doi.org/10.1007/s11771-018-3761-4

[S16] G. Xin, H. Sun, T. Hu, H.R. Fard, X. Sun et al., Large-area freestanding graphene paper for superior thermal management. Adv. Mater. **26**(26), 4521-4526 (2014). https://doi.org/10.1002/adma.201400951

[S17] Z. Ling, C.E. Ren, M.Q. Zhao, J. Yang, J.M. Giammarco et al., Flexible and conductive MXene films and nanocomposites with high capacitance. PNAS **111**(47), 16676-16681 (2014). https://doi.org/10.1073/pnas.1414215111

[S18] L. Chen, X. Shi, N. Yu, X. Zhang, X. Du et al., Measurement and analysis of thermal conductivity of $Ti_3C_2T_x$ MXene films. Materials **11**(9), 1701 (2018). https://doi.org/10.3390/ma11091701

[S19] A. Li, C. Zhang, Y.F. Zhang, Thermal conductivity of graphene-polymer composites: mechanisms, properties, and applications. Polymers **9**(9), 437 (2017). https://doi.org/10.3390/polym9090437

[S20] Y. Li, X. Pei, B. Shen, W. Zhai, L. Zhang et al., Polyimide/graphene composite foam sheets with ultrahigh thermostability for electromagnetic interference shielding. RSC Adv. **5**(31), 24342-24351 (2015). https://doi.org/10.1039/c4ra16421k

[S21] S. Lu, J. Shao, K. Ma, D. Chen, X. Wang et al., Flexible, mechanically resilient carbon nanotube composite films for high-efficiency electromagnetic interference shielding. Carbon **136**, 387-394 (2018). https://doi.org/10.1016/j.carbon.2018.04.086

[S22] D. Zhang, M.G. Villarreal, E. Cabrera, A. Benatar, L.J. Lee et al., Performance study of ultrasonic assisted processing of CNT nanopaper/solventless epoxy composite. Compos. Part B **159**, 327-335 (2019). https://doi.org/10.1016/j.compositesb.2018.10.012

[S23] T. Kuang, L. Chang, F. Chen, Y. Sheng, D. Fu et al., Facile preparation of lightweight high-strength biodegradable polymer/multi-walled carbon nanotubes nanocomposite foams for electromagnetic interference shielding. Carbon **105**, 305-313 (2016). https://doi.org/10.1016/j.carbon.2016.04.052

[S24] H. Wang, K. Zheng, X. Zhang, X. Ding, Z. Zhang et al., 3D network porous polymeric composites with outstanding electromagnetic interference shielding. Compos. Sci. Technol. **125**, 22-29 (2016). https://doi.org/10.1016/j.compscitech.2016.01.007

[S25] Z. Zeng, H. Jin, M. Chen, W. Li, L. Zhou et al., Lightweight and anisotropic porous MWCNT/WPU composites for ultrahigh performance electromagnetic interference shielding. Adv. Funct. Mater. **26**(2), 303-310 (2016). https://doi.org/10.1002/adfm.201503579

[S26] L. Zhang, S. Yang, L. Li, B. Yang, H. Huang et al., Ultralight cellulose porous composites with manipulated porous structure and carbon nanotube distribution for promising electromagnetic interference shielding. ACS Appl. Mater. Interfaces **11**(2), 2559-2559 (2019). https://doi.org/10.1021/acsami.8b21940

[S27] M. Crespo, M. Gonzalez, A.L. Elias, L.P. Rajukumar, J. Baselga et al., Ultra-light carbon nanotube sponge as an efficient electromagnetic shielding material in the GHz range. Phys. Stat. Sol. RRL **8**(8), 698-704 (2014). https://doi.org/10.1002/pssr.201409151

[S28] Z. Chen, C. Xu, C. Ma, W. Ren, H.M. Cheng, Lightweight and flexible graphene foam composites for high-performance electromagnetic interference shielding. Adv. Mater. **25**(9), 1296-300 (2013). https://doi.org/10.1002/adma.201204196

[S29] X. Sun, X. Liu, X. Shen, Y. Wu, Z. Wang et al., Graphene foam/carbon nanotube/poly(dimethyl siloxane) composites for exceptional microwave shielding. Comp. Part A Appl. Sci. Manufact. **85**(199-206), 199-206 (2016). https://doi.org/10.1016/j.compositesa.2016.03.009

[S30] B. Shen, Y. Li, W. Zhai, W. Zheng, Compressible graphene-coated polymer foams with ultralow density for adjustable electromagnetic interference (EMI) shielding. ACS Appl. Mater. Interfaces **8**(12), 8050-8057 (2016). https://doi.org/10.1021/acsami.5b11715

[S31] Y. Wu, Z. Wang, X. Liu, X. Shen, Q. Zheng et al., Ultralight graphene foam/conductive polymer composites for exceptional electromagnetic interference shielding. ACS Appl. Mater. Interfaces **9**(10), 9059-9069 (2017). https://doi.org/10.1021/acsami.7b01017

[S32] W. Song, X. Guan, L. Fan, W. Cao, C. Wang et al., Tuning three-dimensional textures with graphene aerogels for ultra-light flexible graphene/texture composites of effective electromagnetic shielding. Carbon **93**, 151-160 (2015). https://doi.org/10.1016/j.carbon.2015.05.033

[S33] C. Liu, S. Ye, J. Feng, The preparation of compressible and fire-resistant sponge-supported reduced graphene oxide aerogel for electromagnetic interference shielding. Chem. Asian J. **11**(18), 2586-2593 (2016). https://doi.org/10.1002/asia.201600905

[S34] Q. Song, F. Ye, X. Yin, W. Li, H. Li et al., Carbon nanotube-multilayered graphene edge plane core-shell hybrid foams for ultrahigh-performance electromagnetic-interference shielding. Adv. Mater. **29**(31), 1701583 (2017). https://doi.org/10.1002/adma.201701583

[S35] Y. Wan, P. Zhu, S. Yu, R. Sun, C. Wong et al., Ultralight, super-elastic and volume-preserving cellulose fiber/graphene aerogel for high-performance electromagnetic interference shielding. Carbon **115**, 629-639 (2017). https://doi.org/10.1016/j.carbon.2017.01.054

[S36] S.T. Hsiao, C.C.M. Ma, H.W. Tien, W.H. Liao, Y.S. Wang et al., Using a non-covalent modification to prepare a high electromagnetic interference shielding performance graphene nanosheet/water-borne polyurethane composite. Carbon **60**, 57-66 (2013). https://doi.org/10.1016/j.carbon.2013.03.056

[S37] Y. Yang, M.C. Gupta, K.L. Dudley, R.W. Lawrence, Conductive carbon nanofiber–polymer foam structures. Adv. Mater. **17**(16), 1999-2003 (2005). https://doi.org/10.1002/adma.200500615

[S38] Y. Yang, M.C. Gupta, Novel carbon nanotube-polystyrene foam composites for electromagnetic interference shielding. Nano Lett. **5**(11), 2131-2134 (2005). https://doi.org/10.1021/nl051375r

[S39] V. Eswaraiah, V. Sankaranarayanan, S. Ramaprabhu, Functionalized graphene-PVDF foam composites for EMI shielding. Macrom. Mater. Eng. **296**(10), 894-898 (2011). https://doi.org/10.1002/mame.201100035

[S40] H. Zhang, Q. Yan, W. Zheng, Z. He, Z. Yu, Tough graphene-polymer microcellular foams for electromagnetic interference shielding. ACS Appl. Mater. Interfaces **3**(3), 918-924 (2011). https://doi.org/10.1021/am200021v

[S41] D. Yan, P. Ren, H. Pang, Q. Fu, M. Yang et al., Efficient electromagnetic interference shielding of lightweight graphene/polystyrene composite. J. Mater. Chem. **22**(36), 18772-18774 (2012). https://doi.org/10.1039/c2jm32692b

[S42] J. Ling, W. Zhai, W. Feng, B. Shen, J. Zhang et al., Facile preparation of lightweight microcellular polyetherimide/graphene composite foams for electromagnetic interference shielding. ACS Appl. Mater. Interfaces **5**(7), 2677-2684 (2013). https://doi.org/10.1021/am303289m

[S43] Z. Zeng, C. Wang, Y. Zhang, P. Wang, S.I.S. Shahabadi et al., Ultralight and highly elastic graphene/lignin-derived carbon nanocomposite aerogels with ultrahigh electromagnetic interference shielding performance. ACS Appl. Mater. Interfaces **10**(9), 8205-8213 (2018). https://doi.org/10.1021/acsami.7b19427

[S44] Y. Li, B. Shen, X. Pei, Y. Zhang, D. Yi et al., Ultrathin carbon foams for effective electromagnetic interference shielding. Carbon **100**, 375-385 (2016). https://doi.org/10.1016/j.carbon.2016.01.030

[S45] B. Shen, Y. Li, D. Yi, W. Zhai, X. Wei et al., Microcellular graphene foam for improved broadband electromagnetic interference shielding. Carbon **102**, 154-160 (2016). https://doi.org/10.1016/j.carbon.2016.02.040

[S46] B. Shen, W. Zhai, M. Tao, J. Ling, W. Zheng, Lightweight, multifunctional polyetherimide/graphene@Fe_3O_4 composite foams for shielding of electromagnetic pollution. ACS Appl. Mater. Interfaces **5**(21), 11383-11391 (2013). https://doi.org/10.1021/am4036527

[S47] A. Ameli, P.U. Jung, C.B. Park, Electrical properties and electromagnetic interference shielding effectiveness of polypropylene/carbon fiber composite foams. Carbon **60**, 379-391 (2013). https://doi.org/10.1016/j.carbon.2013.04.050

[S48] A. Ameli, M. Nofar, S. Wang, C.B. Park, Lightweight polypropylene/stainless-steel fiber composite foams with low percolation for efficient electromagnetic interference shielding. ACS Appl. Mater. Interfaces **6**(14), 11091-11100 (2014). https://doi.org/10.1021/am500445g

[S49] L. Zhang, M. Liu, S. Roy, E.K. Chu, K.Y. See et al., Phthalonitrile-based carbon foam with high specific mechanical strength and superior electromagnetic interference shielding performance. ACS Appl. Mater. Interfaces **8**(11), 7422-7430 (2016). https://doi.org/10.1021/acsami.5b12072

[S50] F. Moglie, D. Micheli, S. Laurenzi, M. Marchetti, V.M. Primiani, Electromagnetic shielding performance of carbon foams. Carbon **50**(5), 1972-1980 (2012). https://doi.org/10.1016/j.carbon.2011.12.053

[S51] O. Pitkanen, J. Tolvanen, I. Szenti, A. Kukovecz, J. Hannu et al., Lightweight hierarchical carbon nanocomposites with highly efficient and tunable electromagnetic interference shielding properties. ACS Appl. Mater. Interfaces **11**(21), 19331-19338 (2019). https://doi.org/10.1021/acsami.9b02309

[S52] X. Feng, X. Qin, D. Liu, Z. Huang, Y. Zhou et al., High electromagnetic interference shielding effectiveness of carbon nanotube–cellulose composite films with layered structures. Macromol. Mater. Eng. **303**(11), 1800377 (2018). https://doi.org/10.1002/mame.201800377

[S53] Y. Huang, N. Li, Y. Ma, D. Feng, F. Li et al., The influence of single-walled carbon nanotube structure on the electromagnetic interference shielding efficiency of its epoxy composites. Carbon **45**(8), 1614-1621 (2007). https://doi.org/10.1016/j.carbon.2007.04.016

[S54] N. Li, Y. Huang, F. Du, X. He, X. Lin et al., Electromagnetic interference (EMI) shielding of single-walled carbon nanotube epoxy composites. Nano Lett. **6**(6), 1141-

1145 (2006). https://doi.org/10.1021/nl0602589

[S55] Z. Liu, G. Bai, Y. Huang, Y. Ma, F. Du et al., Reflection and absorption contributions to the electromagnetic interference shielding of single-walled carbon nanotube/polyurethane composites. Carbon **45**(4), 821-827 (2007). https://doi.org/10.1016/j.carbon.2006.11.020

[S56] T. Kim, D.D.L. Chung, Mats and fabrics for electromagnetic interference shielding. J. Mater. Eng. Perform. **15**(3), 295-298 (2006). https://doi.org/10.1361/105994906x108594

[S57] M. Bayat, H. Yang, F. Ko, D. Michelson, A. Mei, Electromagnetic interference shielding effectiveness of hybrid multifunctional Fe_3O_4/carbon nanofiber composite. Polymer **55**(3), 936-943 (2014). https://doi.org/10.1016/j.polymer.2013.12.042

[S58] X. Hong, D.D.L. Chung, Carbon nanofiber mats for electromagnetic interference shielding. Carbon **111**, 529-537 (2017). https://doi.org/10.1016/j.carbon.2016.10.031

[S59] A. Gupta, V. Choudhary, Electrical conductivity and shielding effectiveness of poly(trimethylene terephthalate)/multiwalled carbon nanotube composites. J. Mater. Sci. **46**(19), 6416-6423 (2011). https://doi.org/10.1007/s10853-011-5591-8

[S60] M.H. Al-Saleh, U. Sundararaj, Electromagnetic interference shielding mechanisms of CNT/polymer composites. Carbon **47**(7), 1738-1746 (2009). https://doi.org/10.1016/j.carbon.2009.02.030

[S61] Y. Chen, H. Zhang, Y. Yang, M. Wang, A. Cao et al., High-performance epoxy nanocomposites reinforced with three-dimensional carbon nanotube sponge for electromagnetic interference shielding. Adv. Funct. Mater. **26**(3), 447-455 (2016). https://doi.org/10.1002/adfm.201503782

[S62] M.H. Al-Saleh, W.H. Saadeh, U. Sundararaj, EMI shielding effectiveness of carbon based nanostructured polymeric materials: a comparative study. Carbon **60**, 146-156 (2013). https://doi.org/10.1016/j.carbon.2013.04.008

[S63] Z. Zeng, M. Chen, H. Jin, W. Li, X. Xue et al., Thin and flexible multi-walled carbon nanotube/waterborne polyurethane composites with high-performance electromagnetic interference shielding. Carbon **96**, 768-777 (2016). https://doi.org/10.1016/j.carbon.2015.10.004

[S64] M. Arjmand, M. Mahmoodi, G.A. Gelves, S. Park, U. Sundararaj, Electrical and electromagnetic interference shielding properties of flow-induced oriented carbon nanotubes in polycarbonate. Carbon **49**(11), 3430-3440 (2011). https://doi.org/10.1016/j.carbon.2011.04.039

[S65] M. Arjmand, T. Apperley, M. Okoniewski, U. Sundararaj, Comparative study of electromagnetic interference shielding properties of injection molded versus compression molded multi-walled carbon nanotube/polystyrene composites. Carbon **50**(14), 5126-5134 (2012). https://doi.org/10.1016/j.carbon.2012.06.053

[S66] C.C. Wan, J. Li, Graphene oxide/cellulose aerogels nanocomposite: preparation, pyrolysis, and application for electromagnetic interference shielding. Carbhydr. Polym. **150**, 172-179 (2016). https://doi.org/10.1016/j.carbpol.2016.05.051

[S67] P. Ghosh, A. Chakrabarti, Conducting carbon black filled EPDM vulcanizates: assessment of dependence of physical and mechanical properties and conducting character on variation of filler loading. Europ. Polym. J. **36**(5), 1043-1054 (2000). https://doi.org/10.1016/S0014-3057(99)00157-3

[S68] L. Zhang, N.T. Alvarez, M. Zhang, M. Haase, R. Malik et al., Preparation and characterization of graphene paper for electromagnetic interference shielding. Carbon **82**, 353-359 (2015). https://doi.org/10.1016/j.carbon.2014.10.080

[S69] A.A. Eddib, D.D.L. Chung, The importance of the electrical contact between specimen and testing fixture in evaluating the electromagnetic interference shielding effectiveness of carbon materials. Carbon **120**, 337-337 (2017). https://doi.org/10.1016/j.carbon.2017.05.054

[S70] Z. Zeng, C. Wang, G. Siqueira, D. Han, A. Huch et al., Nanocellulose-MXene biomimetic aerogels with orientation-tunable electromagnetic interference shielding performance. Adv. Sci. **7**(15), 2000979 (2020). https://doi.org/10.1002/advs.202000979

[S71] J. Liu, H. Zhang, R.H. Sun, Y. Liu, Z. Liu et al., Hydrophobic, flexible, and lightweight MXene foams for high-performance electromagnetic-interference shielding. Adv. Mater. **29**(38), 1702367 (2017). https://doi.org/10.1002/adma.201702367

[S72] R. Bian, G. He, W. Zhi, S. Xiang, T. Wang et al., Ultralight MXene-based aerogels with high electromagnetic interference shielding performance. J. Mater. Chem. C **7**(3), 474-478 (2019). https://doi.org/10.1039/C8TC04795B

[S73] P. Sambyal, A. Iqbal, J. Hong, H. Kim, M.K. Kim et al., Ultralight and mechanically robust $Ti_3C_2T_x$ hybrid aerogel reinforced by carbon nanotubes for electromagnetic interference shielding. ACS Appl. Mater. Interfaces **11**(41), 38046-38054 (2019). https://doi.org/10.1021/acsami.9b12550

[S74] S. Shi, B. Qian, X. Wu, H. Sun, H. Wang et al., Self-assembly of MXene-surfactants at liquid-liquid interfaces: From structured liquids to 3D aerogels. Angew. Chem. Int. Ed. **58**(50), 18171-18176 (2019). https://doi.org/10.1002/anie.201908402

[S75] H. Xu, X. Yin, X. Li, M. Li, S. Liang et al., Lightweight Ti_2CT_x MXene/poly(vinyl alcohol) composite foams for electromagnetic wave shielding with absorption-dominated feature. ACS Appl. Mater. Interfaces **11**(10), 10198-10207 (2019). https://doi.org/10.1021/acsami.8b21671

[S76] F. Shahzad, M. Alhabeb, C.B. Hatter, B. Anasori, S.M. Hong et al., Electromagnetic interference shielding with 2D transition metal carbides (MXenes). Science **353**(6304), 1137-1140 (2016). https://doi.org/10.1126/science.aag2421

[S77] W. Cao, F. Chen, Y. Zhu, Y. Zhang, Y. Jiang et al., Binary strengthening and toughening of MXene/cellulose nanofiber composite paper with nacre-inspired structure and superior electromagnetic interference shielding properties. ACS Nano **12**(5), 4583-4593 (2018). https://doi.org/10.1021/acsnano.8b00997

[S78] R. Sun, H. Zhang, J. Liu, X. Xie, R. Yang et al., Highly conductive transition metal carbide/carbonitride(MXene)@polystyrene nanocomposites fabricated by electrostatic assembly for highly efficient electromagnetic interference shielding. Adv. Funct. Mater. **27**(45), 1702807 (2017). https://doi.org/10.1002/adfm.201702807

[S79] S. Zhao, H.B. Zhang, J.Q. Luo, Q.W. Wang, B. Xu et al., Highly electrically conductive three-dimensional $Ti_3C_2T_x$ MXene/reduced graphene oxide hybrid aerogels with excellent electromagnetic interference shielding performances. ACS Nano **12**(11), 11193-11202 (2018). https://doi.org/10.1021/acsnano.8b05739

[S80] J. Zhang, N. Kong, S. Uzun, A. Levitt, S. Seyedin et al., Scalable manufacturing of free-standing, strong $Ti_3C_2T_x$ MXene films with outstanding conductivity. Adv. Mater.

32(23), 2001093 (2020). https://doi.org/10.1002/adma.202001093

[S81] Z. Zeng, F. Jiang, Y. Yue, D. Han, L. Lin et al., Flexible and ultrathin waterproof cellular membranes based on high-conjunction metal-wrapped polymer nanofibers for electromagnetic interference shielding. Adv. Mater. **32**(19), 1908496 (2020). https://doi.org/10.1002/adma.201908496

[S82] K. Ji, H. Zhao, J. Zhang, J. Chen, Z. Dai, Fabrication and electromagnetic interference shielding performance of open-cell foam of a Cu–Ni alloy integrated with CNTs. Appl. Surf. Sci. **311**, 351-356 (2014). https://doi.org/10.1016/j.apsusc.2014.05.067

[S83] T. Lee, S. Lee, Y.G. Jeong, Highly effective electromagnetic interference shielding materials based on silver nanowire/cellulose papers. ACS Appl. Mater. Interfaces **8**(20), 13123-13132 (2016). https://doi.org/10.1021/acsami.6b02218

[S84] Z. Zeng, T. Wu, D. Han, Q. Ren, G. Siqueira et al., Ultralight, flexible, and biomimetic nanocellulose/silver nanowire aerogels for electromagnetic interference shielding. ACS Nano **14**(3), 2927-2938 (2020). https://doi.org/10.1021/acsnano.9b07452

[S85] Z. Zeng, W. Li, N. Wu, S. Zhao, X. Lu, Polymer-assisted fabrication of silver nanowire cellular monoliths: Toward hydrophobic and ultraflexible high-performance electromagnetic interference shielding materials. ACS Appl. Mater. Interfaces **12**(34), 38584-38592 (2020). https://doi.org/10.1021/acsami.0c10492

[S86] J. Ma, M. Zhan, K. Wang, Ultralightweight silver nanowires hybrid polyimide composite foams for high-performance electromagnetic interference shielding. ACS Appl. Mater. Interfaces **7**(1), 563-576 (2015). https://doi.org/10.1021/am5067095

[S87] Z. Zeng, M. Chen, Y. Pei, S.I.S. Shahabadi, B.Y. Che et al., Ultralight and flexible polyurethane/silver nanowire nanocomposites with unidirectional pores for highly effective electromagnetic shielding. ACS Appl. Mater. Interfaces **9**(37), 32211-32219 (2017). https://doi.org/10.1021/acsami.7b07643

[S88] Y. Wan, P. Zhu, S. Yu, R. Sun, C. Wong et al., Anticorrosive, ultralight, and flexible carbon-wrapped metallic nanowire hybrid sponges for highly efficient electromagnetic interference shielding. Small **14**(27), 1800534 (2018). https://doi.org/10.1002/smll.201800534

[S89] S. Wu, M. Zou, Z. Li, D. Chen, H. Zhang et al., Robust and stable Cu nanowire@graphene core-shell aerogels for ultraeffective electromagnetic interference shielding. Small **14**(23), 1800634 (2018). https://doi.org/10.1002/smll.201800634

[S90] L. Jia, D. Yan, X. Liu, R. Ma, H. Wu et al., Highly efficient and reliable transparent electromagnetic interference shielding film. ACS Appl. Mater. Interfaces **10**(14), 11941-11949 (2018). https://doi.org/10.1021/acsami.8b00492

[S91] J. Luo, L. Wang, X. Huang, B. Li, Z. Guo et al., Mechanically durable, highly conductive, and anticorrosive composite fabrics with excellent self-cleaning performance for high-efficiency electromagnetic interference shielding. ACS Appl. Mater. Interfaces **11**(11), 10883-10894 (2019). https://doi.org/10.1021/acsami.8b22212

[S92] Y. Wang, F. Gu, L. Ni, K. Liang, K. Marcus et al., Easily fabricated and lightweight PPY/PDA/AgNW composites for excellent electromagnetic interference shielding. Nanoscale **9**(46), 18318-18325 (2017). https://doi.org/10.1039/C7NR05951E

[S93] X. Shui, D.D.L. Chung, Nickel filament polymer-matrix composites with low surface impedance and high electromagnetic interference shielding effectiveness. J. Electr. Mater. **26**(8), 928-934 (1997). https://doi.org/10.1007/s11664-997-0276-4

[S94] L. Li, D.D.L. Chung, Electrical and mechanical-properties of electrically conductive polyethersulfone composites. Composites **25**(3), 215-224 (1994). https://doi.org/10.1016/0010-4361(94)90019-1

[S95] F. Fang, Y. Li, H. Xiao, N. Hu, S. Fu, Layer-structured silver nanowire/polyaniline composite film as a high performance X-band EMI shielding material. J. Mater. Chem. C **4**(19), 4193-4203 (2016). https://doi.org/10.1039/c5tc04406e

[S96] Y. Yu, C.C.M. Ma, C. Teng, Y. Huang, S.H. Lee et al., Electrical, morphological, and electromagnetic interference shielding properties of silver nanowires and nanoparticles conductive composites. Mater. Chem. Phys. **136**(2-3), 334-340 (2012). https://doi.org/10.1016/j.matchemphys.2012.05.024

[S97] N. Zhang, R. Zhao, D. He, Y. Ma, J. Qiu et al., Lightweight and flexible Ni-Co alloy nanoparticle-coated electrospun polymer nanofiber hybrid membranes for high-performance electromagnetic interference shielding. J. Alloys Comp. **784**, 244-255 (2019). https://doi.org/10.1016/j.jallcom.2018.12.341

[S98] N. Zhang, Z. Wang, R. Song, Q. Wang, H. Chen et al., Flexible and transparent graphene/silver-nanowires composite film for high electromagnetic interference shielding effectiveness. Sci. Bull. **64**(8), 540-546 (2019). https://doi.org/10.1016/j.scib.2019.03.028

[S99] M. Arjmand, A.A. Moud, Y. Li, U. Sundararaj, Outstanding electromagnetic interference shielding of silver nanowires: comparison with carbon nanotubes. RSC Adv. **5**(70), 56590-56598 (2015). https://doi.org/10.1039/c5ra08118a

[S100] M.H. Al-Saleh, G.A. Gelves, U. Sundararaj, Copper nanowire/polystyrene nanocomposites: lower percolation threshold and higher EMI shielding. Comp. Part A Appl. Sci. Manuf. **42**(1), 92-97 (2011). https://doi.org/10.1016/j.compositesa.2010.10.003
